# Different association patterns of emotion regulation and heart rate variability in older and younger adults

**DOI:** 10.1093/braincomms/fcaf395

**Published:** 2025-10-10

**Authors:** Kathy Y Liu, Martin J Dahl, Kamen A Tsvetanov, Dorothea Hämmerer, Shai Porat, Clare Loane, Dániel Veréb, Joana B Pereira, Jonathan P Roiser, James B Rowe, Grazia D Femminella, Cam-CAN, Richard N Henson, Lorraine K Tyler, Kamen A Tsvetanov, Carol Brayne, Edward T Bullmore, Andrew C Calder, Rhodri Cusack, Tim Dalgleish, John Duncan, Fiona E Matthews, William D Marslen-Wilson, James B Rowe, Meredith A Shafto, Marta Correia, Karen Campbell, Teresa Cheung, Simon Davis, Linda Geerligs, Rogier Kievit, Anna McCarrey, Abdur Mustafa, Darren Price, David Samu, Jason R Taylor, Matthias Treder, Janna van Belle, Nitin Williams, Daniel Mitchell, Simon Fisher, Else Eising, Ethan Knights, Adam Attaheri, Dace Apsvalka, Maite Crespo-Garcia, Lauren Bates, Tina Emery, Sharon Erzinçlioğlu, Andrew Gadie, Sofia Gerbase, Stanimira Georgieva, Claire Hanley, Beth Parkin, David Troy, Ina Demetriou, Will Duckett, Tibor Auer, Lu Gao, Emma Green, Rafael Henriques, Jodie Allen, Gillian Amery, Liana Amunts, Anne Barcroft, Amanda Castle, Cheryl Dias, Jonathan Dowrick, Melissa Fair, Hayley Fisher, Anna Goulding, Adarsh Grewal, Geoff Hale, Andrew Hilton, Frances Johnson, Patricia Johnston, Thea Kavanagh-Williamson, Magdalena Kwasniewska, Alison McMinn, Kim Norman, Jessica Penrose, Fiona Roby, Diane Rowland, John Sargeant, Maggie Squire, Beth Stevens, Aldabra Stoddart, Cheryl Stone, Tracy Thompson, Ozlem Yazlik, Dan Barnes, Marie Dixon, Jaya Hillman, Joanne Mitchell, Laura Villis, Mara Mather, Robert Howard

**Affiliations:** Division of Psychiatry, University College London, London W1T 7NF, UK; Center for Lifespan Psychology, Max Planck Institute for Human Development, 14195 Berlin, Germany; Leonard Davis School of Gerontology, University of Southern California, Los Angeles, CA 90089, USA; Department of Psychology, University of Cambridge, Cambridge CB2 3EB, UK; Department of Clinical Neurosciences, University of Cambridge CB2 0QQ, Cambridge, UK; Department of Psychology, University of Innsbruck, 6020 Innsbruck, Austria; Institute of Cognitive Neuroscience, University College London, London WC1N 3AZ, UK; Institute of Cognitive Neurology and Dementia Research, Otto-von-Guericke-University Magdeburg, 39120 Magdeburg, Germany; CBBS Center for Behavioral Brain Sciences, 39106 Magdeburg, Germany; German Center for Neurodegenerative Diseases (DZNE), 39120 Magdeburg, Germany; Leonard Davis School of Gerontology, University of Southern California, Los Angeles, CA 90089, USA; Institute of Psychiatry, Psychology and Neuroscience, King’s College London, London SE5 8AB, UK; Department of Clinical Neurosciences, Neuro Division, Karolinska Institutet, 171 76 Stockholm, Sweden; Department of Clinical Neurosciences, Neuro Division, Karolinska Institutet, 171 76 Stockholm, Sweden; Institute of Cognitive Neuroscience, University College London, London WC1N 3AZ, UK; Department of Psychology, University of Cambridge, Cambridge CB2 3EB, UK; Department of Clinical Neurosciences, University of Cambridge CB2 0QQ, Cambridge, UK; Medical Research Council Cognition and Brain Sciences Unit, University of Cambridge, Cambridge CB2 7EF, UK; Institute of Cognitive Neuroscience, University College London, London WC1N 3AZ, UK; Department of Translational Medical Sciences, University of Naples ‘Federico II’, 80131 Naples, Italy; Medical Research Council Cognition and Brain Sciences Unit, University of Cambridge, Cambridge CB2 7EF, UK; Leonard Davis School of Gerontology, University of Southern California, Los Angeles, CA 90089, USA; Division of Psychiatry, University College London, London W1T 7NF, UK

**Keywords:** autonomic, aging, emotion regulation, locus coeruleus, heart rate variability

## Abstract

Several mental health conditions seen in older people are associated with impaired emotion regulation. Heart rate variability (HRV) may be an index of emotion regulation capacity, but it is unclear whether and how aging influences this association. Early neurodegenerative processes, such as Alzheimer’s disease-related reduction of locus coeruleus (LC) integrity, may play a role, as LC modulates both HRV and self-regulatory networks. We pre-registered a cross-sectional study to investigate the relationship between measures of emotion regulation, HRV and LC structural MRI integrity in a lifespan sample of cognitively normal healthy adults (*n* = 678, aged 18–88 years, 51% female), recruited between 2010 and 2012 as part of the Cambridge Centre for Ageing and Neuroscience (Cam-CAN) cohort. We hypothesized that age-related differences in the HRV–emotion regulation relationship could be attributed to reduced LC integrity in older versus younger adults. Exploratory analyses incorporated alternative and novel measures of emotionality and LC rostro-caudal functional connectivity gradients from more recent Cam-CAN studies. In contrast to younger adults, we found an inverse relationship between resting HRV and measures of emotion regulation performance in older adults. There was no evidence that LC integrity influenced this relationship. A more ‘old-like’ LC rostro-caudal functional connectivity gradient, but not LC signal intensity, was related to lower HRV and worse reappraisal outcomes. We identify complexity in the association between HRV and emotion regulation with age and gaps in understanding of the relationship between different measures of LC integrity. Future studies should explore compensatory mechanisms underlying age-related differences in autonomic and emotion regulation.

## Teaser

Liu *et al*. report an inverse association between heart rate variability (HRV) and emotion regulation in older adults, which differed from that of younger adults and was not influenced by locus coeruleus integrity. This opposes the general observation that higher HRV reflects better emotion regulation and supports potential age-related compensatory mechanisms.

## Graphical Abstract

**Graphical Abstract:**
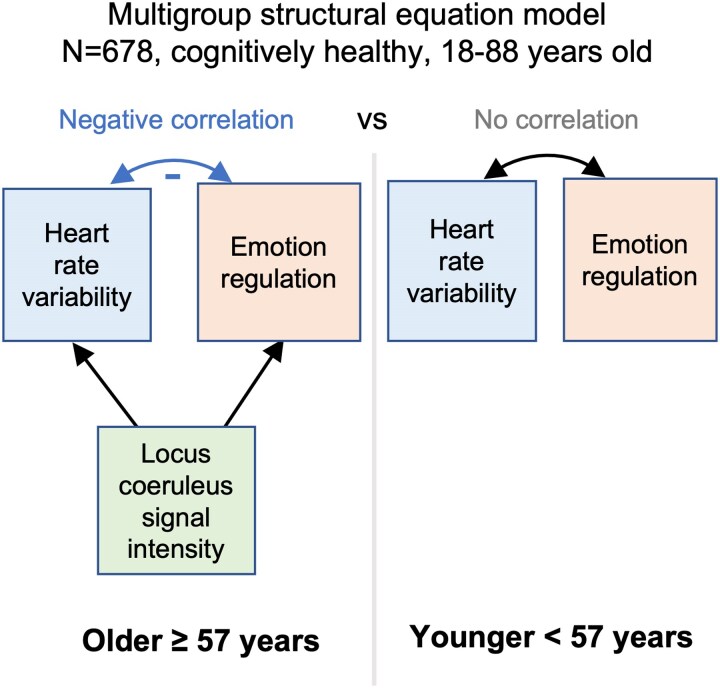

## Introduction

Heart rate variability (HRV), the beat-to-beat variation in heart rate (HR), is considered to index self-regulatory processes such as emotion regulation, which has been described as the neutrally controlled change in one’s cognitive/bodily state from a state that is more intensely emotional to one that is less intensely emotional.^1^ The central autonomic network [comprising insular and prefrontal cortices (PFC), limbic and brainstem regions],^2^ which modulates HRV predominantly through parasympathetic (vagal) activity,^3^ is hypothesized to be integrated with attention and emotion regulation systems to promote adaptive behaviour via inhibitory processes of positive feedback neural circuits.^4,5^ Correspondingly, measures of the integrity and connectivity of these regions have been associated with differences in HRV and emotion regulation capacity^6-9^.

During emotional stress, it is proposed that an adaptive (or maladaptive) decrease in activation of PFC regions leads to reduced tonic inhibition of the amygdala, followed by disinhibition of sympathoexcitatory neurons in the ventrolateral medulla and inhibition of parasympathoexcitatory neurons in the dorsal vagal motor nucleus, which increases HR and decreases resting HRV.^4,10^ Higher HRV measured at rest is consistently associated with, and assumed to be a downstream marker of, better emotion regulation capacity, as measured through task performance^11-14^ and lower levels of psychopathology.^15^ However, accumulating evidence suggests this relationship is actually bidirectional,^16^ as interventions that increase HRV may lead to structural and functional changes within emotion regulation networks^17,18^ and a reduction in depressive symptoms.^19^ Although fewer studies have reported HRV change from baseline to task (reactivity) metrics, a smaller decrease in HRV from baseline to task has generally been associated with better task-based emotion regulation,^12,14^ but findings have been inconsistent.^15^

Given differential age-related trajectories in HRV and emotion regulation constructs, i.e. increasing age has been shown to be associated with improvement in emotion regulation,^20-23^ as well as a decline in HRV,^24,25^ it is unclear whether the positive association between HRV and emotion regulation differs between older (>60 years) and younger adults, as prior studies of HRV–emotion regulation associations have predominantly investigated younger to midlife adults.^12^ In older adults, despite age-related reductions in brain volume^26^ and thickness in association with decline in general cognitive ability,^27^ a stable (or improved) ability to regulate emotion has been linked to altered activity in and function coupling between emotion processing regions such as amygdala and PFC,^28^ as well as preserved cognitive control and executive function.^28^ Age-related decrease in HRV has been associated with lower orbitofrontal cortical thickness^9^ and reduction in PFC functional connectivity, but not structural volume.^29^ Multiple factors likely influence emotion regulation capacity and its measurement in older adults, which may contribute to some of the inconsistencies in the literature.^30,31^ Age-related effects on HRV and emotion regulation may additionally include the earliest stages of neurodegenerative processes such as Alzheimer’s disease,^32^ which can begin up to three decades before dementia onset and underlie subtle preclinical cognitive and behavioural changes.^33^

The locus coeruleus (LC), the earliest site of Alzheimer’s disease-related tau pathology,^34^ plays a central role in regulating arousal and autonomic activity in response to stress^35^ and shows age-related decline in measures of structural and functional integrity from around 60 years,^36-40^ which especially impacts the rostral subregion.^36,41,42^ Different processes underlie the LC MRI signal contrast in younger adults, who show an age-related increase in LC MRI signal intensity up to around 60 years related to neuromelanin accumulation inside LC neurons, in contrast to older adults in whom reductions in LC MRI signal intensity are attributed to age-related LC cell loss^36,38,43,44^ linked to increasing Alzheimer’s disease-related tau lesions.^45,46^ As LC has a sympathoexcitatory and parasympatholytic effect on cardiovascular function through projections to dorsal vagal motor nucleus and ventrolateral medulla neurons, LC activation is expected to result in increased HR and decreased HRV.^35^ Correspondingly, higher HRV has been associated with lower LC integrity, as indexed by decreased LC MRI signal intensity using a neuromelanin-sensitive MRI technique, particularly in older adults,^47,48^ and lower LC fMRI BOLD activity.^49,50^ Given the LC’s role in maintaining cognitive function in aging,^37,51^ reduced LC integrity could be expected to impair emotion regulation processes. It is therefore possible that age-related reduction in LC integrity reverses the direction or reduces the strength of the positive HRV–emotion regulation relationship in older adults. On the other hand, early compensatory processes associated with a relatively more intact LC might impair cognitive control mechanisms in stressful contexts,^52-54^ as higher LC MRI signal intensity in older individuals has been linked to greater stress-related decreases in HRV^48^ and early Alzheimer’s disease-related neuropsychiatric symptoms.^55,56^ Several mental health conditions in older people with and without cognitive impairment are associated with emotion regulation deficits^57-59^. It is important to try to clarify any associations with HRV and LC integrity measures in these individuals, as they may indicate vulnerability for neuropsychiatric symptoms, e.g. agitation, apathy, depression or anxiety, that precede or arise in the context of neurodegenerative conditions such as Alzheimer’s disease.^13,57,60-62^

We investigated the cross-sectional relationship between measures of emotion regulation, HRV and LC MRI integrity in a lifespan sample of cognitively normal healthy adults using the Cambridge Centre for Ageing and Neuroscience (Cam-CAN) dataset, a large population-based cohort aged between 18 and 88 years (*n* = 678). We hypothesized there would be a positive association between HRV and emotion regulation for the whole group, but LC integrity would influence this relationship in older adults. To represent the HRV and emotion regulation constructs, we leveraged the capabilities of structural equation modelling (SEM) to capture the shared variance amongst the observed variables through a composite latent factor, while accounting for measurement error.

## Materials and methods

**Figure 1:**
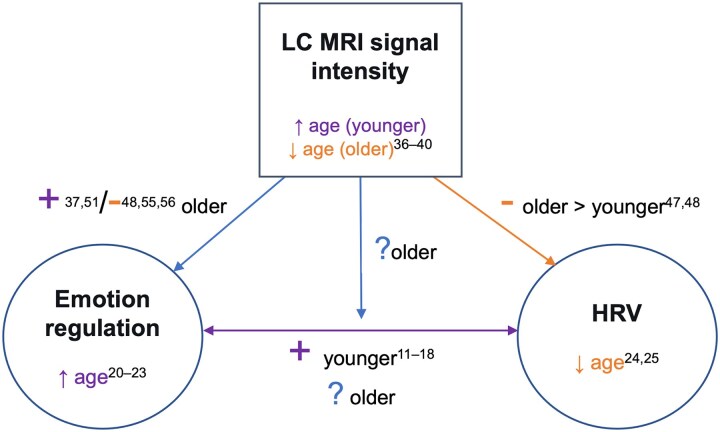
**Proposed relationships between HRV, emotion regulation and LC integrity.** Prior studies have mainly reported negative (−) or positive (+) associations between the variables, as well as age-related differences (increase ↑ or decrease ↓). These include a potential bidirectional, positive relationship between emotion regulation and HRV^11-18^ and a negative association between LC MRI signal intensity and HRV.^47,48^ With older age, studies have reported improvement in emotion regulation^20-23^ and decline in LC integrity^36-40^ and HRV.^24,25^ Unreported or unclear (?) associations (blue arrows) relate to conflicting findings between younger and older adults. We pre-registered the representation of emotion regulation and HRV via latent factors (circles) and LC signal intensity values via observed variables (square). HRV, heart rate variability; LC, locus coeruleus; MRI, magnetic resonance imaging.

The study’s hypotheses and design were pre-registered (https://aspredicted.org/blind.php?x=LXY_VCQ) based on proposed relationships between HRV, emotion regulation and LC integrity (Fig. 1). The pre-registered variables chosen to represent the HRV and emotion regulation constructs are shown in Table 1.

**Table 1:** Pre-registered constructs/variables of interest

Construct/variable	Measures
LC integrity	LC MRI signal intensity (*n* = 605)
HRV	Resting HRV (ECG) (*n* = 439)
	HRV reactivity (PPG) (*n* = 482)
Emotion regulation	Structural MRI volumes (*n* = 653): mPFC, basal forebrain, anterior and posterior insula
	MRI cortical thickness (*n* = 653): lateral orbitofrontal cortex
	Resting state functional connectivity^a^ (*n* = 652): amygdala-mPFC, *LC-mPFC* and *LC-amygdala*
	Diffusion MRI (*n* = 641): *dorsal noradrenergic bundle (LC-transentorhinal cortex)*
	*Emotion regulation task score* (*n* = 290)

dmPFC, dorsomedial PFC; ECG, electrocardiography; HRV, heart rate variability; LC, locus coeruleus; mPFC, medial prefrontal cortex; PPG, photoplethysmography; vmPFC, ventromedial PFC. Pre-registered primary and secondary (italicized font) measures are shown. For mPFC functional connectivity, mPFC was defined as vmPFC or dmPFC. ^a^ Amygdala-whole brain resting state functional connectivity was initially pre-registered as a primary emotion regulation measure, but was subsequently considered to be insufficiently specific and was not analysed.

### Participants

A healthy, population-based adult lifespan sample (target *n* = 700) was recruited between 2010 and 2012 as part of the Cam-CAN project. Data used in the preparation of this work were obtained from the Cam-CAN repository (available at http://www.mrc-cbu.cam.ac.uk/datasets/camcan/), and details of the Cam-CAN study protocol have been published previously.^63,64^ Participants were excluded based on the following criteria: Mini Mental State Examination (MMSE) < 25; failing to hear a 35 dB 1 kH tone in either ear; poor English language skills (non-native or non-bilingual speakers); self-reported substance abuse and serious health conditions (e.g. major psychiatric conditions, or a history of stroke or heart conditions) or MRI or MEG contraindications (e.g. ferromagnetic metallic implants, pacemakers or recent surgery). See Supplementary Table 1 for MRI acquisition details. Ethical approval for the study was obtained from the Cambridgeshire 2 (now East of England—Cambridge Central) Research Ethics Committee (reference: 10/H0308/50), and all participants provided written informed consent prior to the study.

### HRV indices

Electrocardiogram (ECG) data were obtained with a sampling frequency of 1 kHz during the 8 min 40 s resting state with eyes closed magnetoencephalography (MEG) recording session.^64^ Photoplethysmography (PPG) data were obtained from a pulse oximeter placed on the left index finger, sampled at 50 Hz, in a functional MRI session during resting state with eyes closed (8 min 40 s), followed by watching a compelling and unfamiliar movie, an excerpt of Alfred Hitchcock's ‘Bang! You're Dead’ (8 min).^64^

The ECG/PPG data were processed using PhysioNet Cardiovascular Signal Toolbox^65,66^ in MATLAB (The MathWorks Inc., MATLAB version 2019b). Mean HRV indices for each participant were calculated across sliding 5 min windows in 30 s increments for the duration of the ECG waveform. Of 646 subjects with ECG channel recordings, *n* = 439 had usable ECG data after a signal quality index threshold of 0.3 was applied, and segments classified as atrial fibrillation were excluded (any participant with >50% atrial fibrillation was excluded). As the signal quality of the PPG was lower than the ECG data, the atrial fibrillation detection setting was not applied, as this excluded a substantial proportion of segments (resulting in only *n* = 69 usable observations from PPG data recorded during movie watching). In total, *n* = 482 had usable PPG data during resting state, and *n* = 462 had usable PPG data during movie-watching.

We used the root mean square of successive differences (RMSSD) in normal-to-normal (NN) intervals and high frequency (HF) (0.15 Hz ≤ HF < 0.4 Hz) band HRV metrics, which are time- and frequency-domain HRV indices more specific to parasympathetic (vagal) function.^14,67^ The HRV metrics (RMSSD and HF) were log-transformed to obtain more normal distributions. HRV during rest and HRV reactivity measures were obtained, the latter by calculating the difference between mean HRV indices (via PPG) during movie watching and rest. HR was calculated as the mean number of NN intervals within each 60 s interval.

### LC integrity measures

LC MRI signal intensity values were acquired from an earlier study^36^ using the Cam-CAN MT-weighted scans (see Supplementary Table 1 for MRI acquisition details). In summary, single-subject MT-weighted images were upsampled to 0.8-mm isotropic resolution, bias-corrected and spatially normalized to a studywise space using ANTS v1.2 software package. The LC mask, defined as the conjunction of labelled voxels from two independent manual segmentations on the studywise MT-weighted template, was warped back to the individual native space. Individual scans were inspected for motion artefacts, leading to exclusion of 18 participants (age range 20–80 years, mean [SD] = 59 [22] years). Mean LC signal intensity values from the remaining 605 participants (97% of the original sample) were normalized to a pontine reference region to calculate each individual’s contrast ratio (LC CR), using the formula below, which was included in the subsequent analysis:


$$\mathrm{LC}\;\mathrm{CR}=\frac{\left(S_{\mathrm{LC}}-S_{\mathrm{ref}}\right)}{S_{\mathrm{ref}}}$$

where *S*_LC_ represents the signal intensity of the LC and *S*_ref_ the signal intensity of the pontine reference region.

For secondary analyses, each participant’s LC was divided into a rostral and caudal region either side of the median voxel along the *z*-axis of the individual’s LC to obtain mean rostral and caudal LC CR values for each participant.

In light of a relevant recent publication (published after the pre-registration of our study) showing age-related differences in LC functional rostro-caudal functional connectivity gradients in the Cam-CAN sample, where a more ‘old-like’ functional gradient (corresponding to a loss of rostral-like connectivity, increased asymmetry and more clustered functional organization of the LC observed in older participants) was associated with worse negative reappraisal performance,^68^ we also decided to include these individual LC functional gradients as exploratory measures. This analysis was therefore not pre-registered. In this resting-state fMRI study, the LC was masked for each subject and spatial smoothing was applied with a 3 mm full width half maximum (FWHM) isotropic 3D Gaussian kernel within the mask. To obtain participant-level measures representing the LC functional gradient, nine spatial model parameters describing the individual gradient spatial layout were obtained. These were three orders corresponding to different spatial features (first order, quadratic, cubic) for each MNI axis (*x*, *y*, *z*). The two parameters of interest were the first and cubic order coefficients of the spatial model describing the connectivity profile variation along the LC longitudinal axis (MNI *z*-axis). Lower first and higher cubic order parameters describing spatial features of the gradient along the *z*-axis were associated with a ‘young-like’ gradient, whereas higher first and lower cubic order parameters of this gradient were associated with an ‘old-like’ gradient.

### Emotion regulation indices

The selection of pre-registered primary neural indices of emotion regulation was based on published correlates of central autonomic and emotion regulation, and included medial prefrontal cortex (mPFC),^2,6,8^ basal forebrain^2^ and insula^2,6^ grey matter (GM) volumes, lateral orbitofrontal cortical thickness^7^ and resting state amygdala-mPFC functional connectivity.^69^ Additional pre-registered secondary measures considered anatomically and/or functionally relevant included LC-mPFC resting state functional connectivity,^35^ dorsal noradrenergic ascending bundle integrity^70^ and cognitive reappraisal as a psychological index of emotion regulation,^12,71^ which involves reinterpretation of the stimulus to change one’s emotional response to it.^72,73^ Cognitive reappraisal for low intensity emotions shows a stronger association with resting-state functional connectivity measures compared to high intensity emotions, possibly reflecting the more flexible recruitment of cognitive resources for regulating high intensity emotions.^74^ Emotion regulation strategies such as cognitive reappraisal may be more impaired in older versus younger adults due to age-related decline in cognitive control and cortical structural integrity.^20^

### Regional GM volume and cortical thickness measures

See Supplementary Table 1 for MRI acquisition details. T1-weighted structural images were processed using the Computational Anatomy Toolbox (CAT12) (https://neuro-jena.github.io/cat//) in Statistical Parametric Mapping (SPM12) software (https://www.fil.ion.ucl.ac.uk/spm/). The CAT12 default voxel-based morphometry (VBM) pre-processing pipeline resulted in normalized and modulated images registered to a template space, segmented into GM, white matter (WM) and cerebrospinal fluid (CSF). Total intracranial volume (TIV) was estimated using the CAT12 ‘Estimate TIV’ function. Mean region-of-interest (ROI) GM volumes for each participant were estimated using the Neuromorphometrics atlas (http://Neuromorphometrics.com/). Bilateral regional volume values for anterior and posterior insula, basal forebrain and medial frontal cortex (*n* = 653) were extracted and divided by TIV for subsequent statistical analysis. The CAT12 preprocessing pipeline also included a surface-based morphometry (SBM) analysis that estimated ROI cortical thickness values using the Desikan–Killiany (DK40) atlas,^75^ from which left and right lateral orbitofrontal cortical thickness measures (*n* = 653) were extracted for statistical analysis.

### Resting state functional connectivity measures

See Supplementary Table 1 for MRI acquisition details. The fMRI images (*n* = 652) were preprocessed using the CONN toolbox v.20b^76^ default preprocessing pipeline in SPM12 software (https://www.fil.ion.ucl.ac.uk/spm/). This included functional realignment and unwarping, slice-timing correction, outlier identification using Artifact Detection Tools (ART, https://www.nitrc.org/projects/artifact_detect, which flagged acquisitions with framewise displacement above 0.9 mm or global BOLD signal changes above 5 SD as potential outliers), normalization into standard Montreal Neurologic Institute (MNI) space, segmentation into GM, WM and CSF tissue classes, and functional smoothing with a Gaussian kernel of 8 mm FWHM. The CONN toolbox default denoising pipeline was then used to minimize the influence of artifactual factors on functional connectivity measures. This included removal via linear regression of potential confounding effects, i.e. noise components from WM and CSF areas,^77^ estimated subject-motion parameters,^78^ and identified outlier scans or scrubbing,^79^ from the BOLD signal. Temporal band-pass filtering then removed temporal frequencies below 0.008 Hz or above 0.09 Hz from the BOLD signal, to minimize the influence of physiological, head-motion and other noise sources.

As mPFC shows differential connectivity patterns and functionally distinct subregions underlying a number of psychological processes, we focused on the ventromedial PFC (vmPFC) and dorsomedial PFC (dmPFC) regions to investigate emotion regulation-related mPFC functional connectivity, as these regions are proposed to be involved in reappraisal and HRV regulation.^8,29^ Thus, four bilateral ROIs were used for ROI-to-ROI connectivity analyses: vmPFC, dmPFC, amygdala and LC. The amygdala ROI was defined by the Harvard-Oxford atlas, the LC ROI mask (available from https://osf.io/r2bwk/)^80^ was obtained from a previous study in Cam-CAN participants,^36^ and vmPFC and dmPFC ROI masks (available from https://identifiers.org/neurovault.collection:819) were meta-analytically defined regions from earlier resting state analyses.^81^ ROI-to-ROI *z*-scores for each subject (*n* = 652) were extracted via Fisher transformation of the pairwise temporal correlations (Pearson’s correlation coefficients, *r*) computed across all pairs of ROIs (vmPFC-right/left amygdala, dmPFC-right/left amygdala, vmPFC-LC, dmPFC-LC and LC-right/left amygdala).

### Diffusion tensor imaging measures

See Supplementary Table 1 for MRI acquisition details. As tau pathology is identified earliest in the LC followed by the transentorhinal cortex (TEC),^34^ the structural integrity of the LC-TEC fibre pathway is proposed to reflect the earliest brain connectivity changes due to Alzheimer’s disease-related tau pathology. We extracted diffusion tensor imaging (DTI) metrics within this ascending noradrenergic pathway bundle using a probabilistic atlas of the LC-TEC pathway for each hemisphere^70^ with the MRtrix3 software package v. 3.0.3.^82^ After denoising and removal of Gibbs ringing artefacts, eddy current and motion correction and bias field correction were applied to participants’ DWI data. Whole brain maps of tensor-derived parameters were generated from diffusion tensor estimates via a binary mask image for each subject’s DWI data. Using ANTS v2.3.5 software, the right and left LC-TEC atlases were resampled to 1 mm MNI space, then backwarped to individual subject space using MNI-to-tensor image transformations with nearest neighbour interpolation. Mean values for fractional anisotropy, radial diffusivity and mean diffusivity were extracted from the binarized, warped atlas ROIs for each participant (*n* = 641).

### Emotional reactivity and regulation task measures

The Cam-CAN ERRT was a film-based paradigm.^83^ Participants rated their emotional responses, i.e. how negative or positive they felt on a scale from 1 (not at all) to 11 (extremely), after viewing a series of 40 film clips of 30 s that were either positive (e.g. infants laughing), neutral (e.g. weather report) and negative (e.g. documentary of the Rwandan genocide) in valence. Before each film clip, participants received a prompt to indicate the valence and viewing instruction for the clip (e.g. WATCH NEUTRAL or REGULATE NEGATIVE). Here, participants were instructed either to watch the film clip and allow themselves to feel any emotions that naturally arose without trying to deliberately distract themselves from its content or effortfully regulate their emotions in any way (WATCH), or, for half of the negative film clips, participants were explicitly asked to try to reduce (downregulate) any unwanted distressing affect in response to the film by changing the way they thought about its content (REGULATE). Thus, there were positive and negative ratings for the four conditions (watch neutral, watch positive, watch negative and reappraise negative). After watching the film clip, participants were visually presented with a 10-point scale to rate both their negative and positive affective reactions experienced during the clip and to rate how much they simply watched the clip versus regulated their affect as a measure of compliance. Each condition was presented twice, with four trials in each block. Emotional blocks were followed by 45 s washout clips, i.e. a calming film clip (e.g. waves on a beach with a meditative soundtrack), to return affective levels to prestimulus baseline. Films were randomized across the WATCH and REGULATE conditions separately for each participant, and the presentation order of condition was pseudo-randomized, starting with a neutral block and ending with a positive block. The study from which the film-based paradigm was derived reported matching all aversive films on emotionality based on independent ratings by assessors blind to experimental condition.^83,84^

After ratings were scaled from negative to positive, a ‘negative reappraisal’ score was calculated by subtracting negative affectivity ratings after watching negative films from negative affectivity ratings after reappraising negative films. We used this score to represent the ability to effortfully downregulate negative affect using reappraisal, with higher scores representing better emotion regulation. Of *n* = 316 available ERRT scores, *n* = 26 were excluded due to session notes indicating that they did not understand the task, or having scores >3 SD from the mean, resulting in *n* = 290 observations.

After our analysis was pre-registered, a study using the Cam-CAN ERRT data was published, which identified four theoretically plausible latent factors explaining the ERRT ratings, which were basal negative affect (negativity derived from neutral and positive stimuli), positive regulation (positivity derived from negative stimuli), negative reactivity (negativity derived from negative stimuli) and positive reactivity (positivity derived from neutral and positive stimuli).^83^ We therefore additionally included this four-factor latent model as an exploratory analysis approach, which was not pre-registered, as it potentially provides additional information for age-related analyses of emotionality in addition to the single bivalence scales.

### Statistical analyses

To conduct SEM, all observed continuous variables were scaled to have a mean of 5 and a standard deviation (SD) of 2 to improve model convergence. Model fit was evaluated using the root-mean-square error of approximation (RMSEA), the comparative fit index (CFI) and the standardized root mean residual (SRMR). Good model fit was defined as RMSEA < 0.06 (acceptable: 0.06–0.08); CFI > 0.95 (acceptable: 0.90–0.95) and SRMR < 0.08 (acceptable: 0.08–0.10).^85^ All models were estimated using the lavaan package, version 0.6-12,^86^ in R,^87^ version 3.5.161, using all available data via full information maximum likelihood estimation and the robust maximum likelihood estimator with a Yuan–Bentler scaled test statistic.

Confirmatory factor analyses (CFAs) were conducted to validate the pre-registered latent factor models for HRV and primary (+/− secondary) measures of emotion regulation. HR was included as a covariate loading onto HRV in the statistical models to adjust HRV for HR, as this has been reported to influence the cardiovascular predictive value of HRV and its reproducibility,^88^ and they have different neural components at rest.^29^ To manage the high collinearity between pairs of HF and RMSSD measures that limited the fit of the pre-specified HRV latent factor model in the initial CFA (Supplementary Fig. 1), the standardized scores were averaged to represent vagally mediated HRV, resulting in one ECG (resting) and two PPG (resting and movie-watching) HRV variables. The averaged HRV variables are unitless and reflect the central tendency of the standardized scores of HF and RMSSD. To improve model fit, we tested the impact of removing any variables with low standardized factor loadings (<0.3) and examined theoretically plausible modification indices. We report in Supplementary Methods 1 and 2 how more optimal models were explored when the *a priori* measurement models showed inadequate fit indices for the data.

To test our pre-registered primary hypothesis that there was a positive association between HRV and emotion regulation capacity, we investigated the covariance (correlation) between HRV and emotion regulation latent factors. Our pre-registered secondary hypothesis was that LC integrity moderates the relationship between HRV and emotion regulation, particularly in older adults in whom a functionally relevant age-related reduction in LC integrity is presumed to occur. However, after we accessed and started to analyse the data, we considered that our ability to perform and interpret a moderation analysis was limited. This was related to the final sample size providing insufficient power for a cross-sectional interaction analysis, particularly for older adults (see Supplementary Results 1) and, as we did not have a strong hypothesis for the directionality of effects due to evidence for a bidirectional relationship between HRV and emotion regulation, the cross-sectional nature of the Cam-CAN data would be unable to shed light on the direction of influence of any LC moderation. As an alternative, we aimed to understand the role of LC integrity within a bidirectional relationship between HRV and emotion regulation by assessing direct regression paths of LC on HRV and emotion regulation in older adults. We also examined whether the addition of LC integrity as a covariate in the correlational models could account for any HRV–emotion regulation associations, over and above the effect of age, which could support (but not rule out) the potential presence of a moderation effect.

We compared the covariance structure between younger and older adults using a multigroup analysis, defining the age breakpoint based on LC MRI signal intensity differences identified in a previous study based on the Cam-CAN cohort,^36^ resulting in a younger (<57 years) and older (≥57 years) age group. This age breakpoint was pre-registered as it was considered to represent the age at which age-related LC cell loss begins to be detected in older adults in this study population. Age was also included as a covariate in the models to statistically control for age differences across the whole sample and within younger/older groups. Other potential confounds were included as additional covariates loading onto HRV and emotion regulation variables in the correlational model, which were participant sex, cardiovascular health status (total number of conditions present out of diabetes, stroke, hypertension, high cholesterol, myocardial infarction), age of completing full time education, beta-blocker medication status (present/absent) and fluid intelligence (as this shows age-related decline^89,90^ and was measured by the Cattell Culture Fair test total score^91^). Age was allowed to regress onto beta-blocker medication status, fluid intelligence and cardiovascular health status. As secondary analyses, we tested for LC MRI signal intensity subregional effects by using only rostral or caudal values.

For factor loadings, covariances and regressions, we formally assessed the statistical significance of individual parameters of interest within a model using the likelihood ratio test (LRT), which involved comparing the fit of a model with the parameter of interest freely estimated to a nested model with the same parameter fixed to zero. Similarly, we also used the LRT to test the statistical significance of differences in parameters of interest between younger and older age groups, by comparing a model where the parameter was constrained to be equal to one where it was freely estimated across groups. For the multigroup models, measurement invariance across age groups was evaluated using LRTs to establish equal loadings across groups, i.e. metric invariance.^92^

## Results

### Descriptive statistics

Table 2 shows descriptive statistics of the HRV, primary emotion regulation and LC variables, covariates, and the extent of missing observations in the younger (<57 years, *n* = 364) and older (≥57 years, *n* = 314) subgroups. Additional descriptive statistics [scaled values mean (SD), skewness and kurtosis] are reported in Supplementary Table 2. Descriptive statistics for the secondary emotion regulation measures are shown in Supplementary Table 3, and pairwise correlations between all analysed variables of interest for the whole sample and both age subgroups are shown in Supplementary Tables 4–6.

**Table 2:** Descriptive statistics for HRV, primary emotion regulation and LC variables and demographic covariates

Variable	No. of observations (% of total)		Mean (SD) or *Proportion*		
	Younger group	Older group	Younger group	Older group	*P*-value^f^ (Cohen’s *d*)
Age	364 (100)	314 (100)	39.8(10.3) years	71.9 (8.4) years	2.2e−16 (3.41)
% Female	364 (100)	314 (100)	*51.9* (*189/364)*	*49.0* (*154/314)*	0.50
Age when completed full time education	355 (97.5)	313 (99.7)	21.2 (3.2) years	19.6 (4.7) years	1.47e−10 (−0.40)
Cattell fair total score	349 (95.9)	303 (96.5)	27.4 (4.9)	18.8 (6.7)	2.2e−16 (−1.48)
Cardiovascular health status score^a^	359 (98.6)	308 (98.1)	0.1 (0.4)	0.7 (0.9)	2.2e−16 (0.88)
% Beta-blocker drug present^b^	364 (100)	314 (100)	*0.8* (*3/364)*	*5.7* (*18/314)*	2.19e−04 (0.29)
Negative reappraisal score	160 (44.0)	130 (41.4)	−0.008 (1.07)	−0.018 (1.12)	0.97
logHRV^c^ (resting ECG)	259 (71.2)	180 (57.3)	5.17 (0.8)	4.7 (1.2)	2.54e−05 (−0.42)
logHRV^c^ (resting PPG)	258 (70.9)	224 (71.3)	6.9 (0.7)	6.8 (0.7)	0.48
logHRV^c^ (movie PPG)	246 (67.6)	216 (68.8)	6.9 (0.7)	6.9 (0.7)	0.99
VBM mPFC^d^	347 (95.3)	306 (97.5)	0.0012 (1.37e−04)	0.0010 (1.38e−04)	<2.2e−16 (−1.27)
VBM basal forebrain^d^	347 (95.3)	306 (97.5)	0.00043 (3.80e−05)	0.00038 (3.88e−05)	<2.2e−16 (−1.18)
VBM insula^d^	347 (95.3)	306 (97.5)	0.0023 (2.01e−04)	0.0020 (1.89e−04)	<2.2e−16 (−1.14)
SBM lateral OFC	347 (95.3)	306 (97.5)	2.94 (0.15) mm	2.82 (0.18) mm	<2.2e−16 (−0.75)
Mean LC CR^e^	329 (90.4)	276 (87.9)	0.09 (0.02)	0.10 (0.03)	1.99e−09 (0.48)

HRV, heart rate variability; LC CR, locus coeruleus contrast ratio; mPFC, medial prefrontal cortex; OFC, orbitofrontal; SBM, surface-based morphometry; VBM, voxel-based morphometry.

Additional descriptive statistics [scaled values mean (SD), skewness and kurtosis] are reported in Supplementary Table 2. ^a^Cardiovascular health status score was the total number of conditions present out of diabetes, stroke, hypertension, high cholesterol, myocardial infarction. ^b^Beta-blocker drug status was a binary measure (absent/present). ^c^LogHRV values were derived by averaging logHF and logRMSSD scores, which were highly correlated (*r* > 0.9). ^d^Brain volumes were adjusted for (divided by) total intracranial volume. ^e^LC MRI signal intensities were normalized to a pontine reference region. ^f^*P*-values reflect group comparisons of unscaled mean values based on Welch’s *t*-tests or Mann–Whitney U-tests (for MMSE, Cattell Fair score) for approximately normally distributed continuous variables and Mann–Whitney U-tests for non-normally distributed variables based on skewness (>±1) and/or kurtosis (>3). Categorical comparisons were tested using *χ*^2^ (% female) or Fisher’s exact tests (% beta-blocker). If there was a significant between-group difference, the size of the difference (Cohen’s *d*) is reported.

### HRV and emotion regulation measurement models

**Figure 2:**
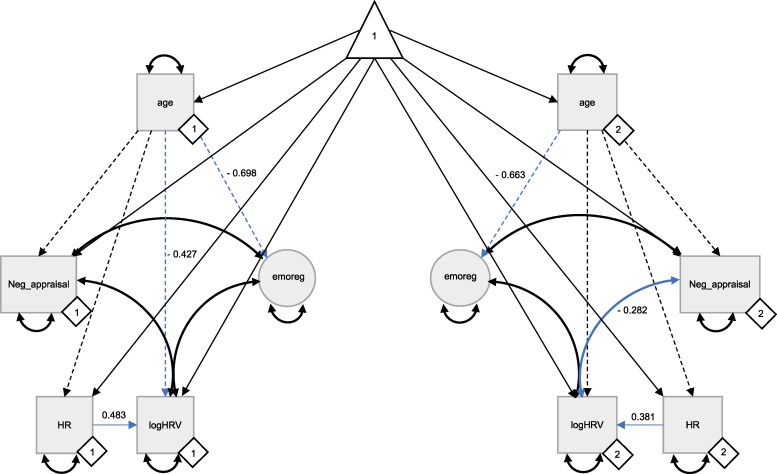
**Correlational multigroup SEM for emotion regulation capacity and resting HRV.** A multigroup SEM showed a negative covariance between *HR*-adjusted resting HRV obtained using ECG data (*logHRV*) and *Negative Reappraisal (Neg_reappraisal)* that was driven by older adults, which survived adjustment for age. The primary +/− secondary variables loading onto the ‘neural’ emotion regulation latent factor (*emoreg*) have not been shown for clarity. The constant is represented by a triangle, latent factors are represented by circles and observed variables are represented by squares/rectangles. Only significant standardized covariances (double-headed arrows) and regressions (single-headed arrows), formally assessed using the LRT, are reported and shown in blue. Number of observations used: *n* = 364 (11 missing patterns) younger adults, *n* = 314 (10 missing patterns) older adults. Younger (left side; ♢1) and older (right side; ♢2) adult submodels are shown. The multigroup structural equation model for HRV reactivity using PPG data showed no significant covariances between the PPG HRV latent change score (*dhrv1*) and emotion regulation capacity, and multigroup analysis was not subsequently conducted for this model as it did not achieve metric invariance. Further information on covariance/regression estimates and model fit indices are reported in Supplementary Table 7. ECG, electrocardiogram; HR, heart rate; HRV, heart rate variability; PPG, photoplethysmography; SEM, structural equation model.

After observing a poor correlation between ECG and PPG-derived resting HRV (*r*  *=* 0.27), differential relationships with age, and considering evidence for higher accuracy and reliability of ECG measures compared to fingertip PPG,^93,94^ we decided to investigate resting HRV (via ECG) and HRV reactivity (via PPG) in separate correlational models (Supplementary Methods 1 and Fig. 2), with HRV reactivity (via PPG) most optimally represented in a latent change score model (ΔHRV)^95^ (Supplementary Fig. 2).

The pre-registered emotion regulation latent factor showed good fit (Supplementary Fig. 3), although resting state functional connectivity variables and the negative reappraisal task score showed low (<0.1) standardized factor loadings. As removal of only the latter improved model fit (CFI = 0.994, RMSEA = 0.022, SRMR = 0.022), we distinguished between ‘neural’ and ‘psychological’ emotion regulation capacity and subsequently analysed these constructs separately in the correlational model (Fig. 2).

### Correlations between HRV and emotion regulation capacity

Following the CFAs above, we assessed covariances between resting HRV (via the single averaged ECG measure) or HRV reactivity (via the PPG latent change score) and ‘neural’ (via the emotion regulation latent factor comprising primary +/− secondary structural/functional measures) and ‘psychological’ (via the negative reappraisal task score) emotion regulation (Fig. 2). Full details for all unadjusted and age-adjusted multigroup correlational models, including standardized covariance and age regression estimates for the main parameters of interest and fit indices, are shown in Supplementary Table 7.

We found a negative correlation (i.e. standardized covariance) between resting HRV and the negative reappraisal task score across the whole sample, which survived adjustment for age (*χ*^2^_diff_ = 7.40, df_diff_ = 1, *P* = 0.007). Multigroup analysis revealed that this was driven by a significant negative correlation in older adults (*χ*^2^_diff_ = 11.85, df_diff_ = 1, *P* < 0.001), which was significantly different to the same path in younger adults (*χ*^2^_diff_ = 6.88, df_diff_ = 1, *P* = 0.009), in whom the association was not significant (*χ*^2^_diff_ = 0.50, df_diff_ = 1, *P* = 0.48). There was also a positive correlation between resting HRV and the latent ‘neural’ emotion regulation factor across the whole sample that was accounted for by negative associations between these variables and age, which was driven by younger adults (Supplementary Table 7). No significant correlations were observed between emotion regulation capacity and HRV reactivity measured using PPG, and this model did not achieve full metric invariance for multigroup analysis.

Analogous to earlier observations, in a subsequent exploratory analysis of covariances between HRV and four previously identified latent emotionality factors in a model that incorporated all emotional reactivity and regulation task (ERRT) score variables^83^ (Supplementary Fig. 4), only the association between resting HRV and the positive regulation latent factor was significantly different between age groups after adjustment for age (*χ*^2^_diff_ = 8.536, df_diff_ = 1, *P* < 0.003) and was significantly negative in older adults (*χ*^2^_diff_ = 4.8845, df_diff_ = 1, *P* = 0.0271) and positive in younger adults (*χ*^2^_diff_ = 4.2962, df_diff_ = 1, *P* = 0.0382). Across the whole sample, age accounted for negative covariances between HRV and the positive and negative reactivity and positive regulation latent emotionality factors (Supplementary Table 7). Again, no significant associations were found between the four emotionality factors and PPG HRV reactivity.

Taken together, older adults showed a negative association between resting HRV and emotion regulation outcomes, and this relationship significantly differed from that seen in younger adults.

### The influence of LC measures and other covariates on the HRV–emotion regulation association in older adults

The negative relationship between resting HRV and emotion regulation outcomes in older adults persisted after the addition of LC MRI signal intensity or LC gradient measures as a covariate in the age-adjusted correlational models.

When we inspected regressions of LC integrity on HRV and emotion regulation outcomes, we observed that age accounted for the negative association observed between LC MRI signal intensity (including subregional) measures and HRV for the whole sample and younger subgroup. In comparison, participant-level LC gradient measures did not show associations with HRV, although a windowed approach revealed that a more ‘old-like’ LC gradient was associated with lower HRV, above the effects of age, sex, education, LC MRI signal intensity and fluid intelligence (for the whole sample: partial adjusted *R*^2^ = 0.15, *P* < 0.019; for adults >50 years: partial adjusted *R*^2^ = 0.33, *P* < 0.036). A more ‘old-like’ LC functional gradient, represented by participant-level higher first order and lower cubic order coefficients of the individual LC gradient spatial layout along its longitudinal axis (MNI *z*-axis), was also associated with worse negative reappraisal performance for the whole sample and older subgroup, above the effects of age, in line with previous findings using a windowed approach.^68^ Similarly, in the four-factor latent model, a more ‘old-like’ LC gradient was associated with lower positive regulation in the whole sample and younger adult subgroup.

The age-adjusted negative association between HRV and successful reappraisal outcomes in older adults was robust and remained significantly different from younger adults after the inclusion of participant sex, age at full time education completion, fluid intelligence scores, beta-blocker medication and cardiovascular health status. However, the positive association between HRV and the positive regulation factor in younger adults no longer remained statistically significant after inclusion of the additional covariates.

## Discussion

In contrast to the hypothesized positive relationship between emotion regulation and HRV across the lifespan sample (*n* = 678, 18–88 years), we found a negative correlation between resting HRV and successful emotion regulation performance that was driven by older adults, which differed significantly from the relationship in younger adults. We did not find evidence to support our hypothesis that LC integrity accounted for this association in older adults above the effects of age, although there was evidence that a more ‘old-like’ LC functional gradient was related to worse emotion regulation performance and lower HRV.

Our findings in older adults are opposite to the general observation that higher HRV is a marker of better self-regulatory capacity. Aging is associated with abnormalities in the central and peripheral regulation of autonomic function, along with compensatory changes,^96^ which increases the complexity of the relationship between autonomic and emotion regulation in older adults. Earlier studies have also reported a possible non-linear relationship between cardiac parasympathetic control and well-being, where very high and very low levels are less adaptive versus moderate levels.^97,98^ Higher resting HRV in older adults might reflect lower arousal levels with reduced cognitive and self-regulatory functioning. Alternatively, compensatory changes in the central autonomic network with increasing age^99^ or Alzheimer’s disease-related neuropathology^100^ could explain the observed inverse HRV–emotion regulation relationship in older adults. In support of this, our findings align with a recent study reporting that higher HRV was associated with agitation risk in Alzheimer’s disease,^101^ indicating a need to further investigate how Alzheimer’s disease-related compensatory activity might alter autonomic and emotion regulation processes, which may support their potential as therapeutic targets for neuropsychiatric symptoms.

Our findings were specific to psychological measures of emotion regulation, as the positive association between the latent ‘neural’ emotion regulation factor and resting HRV was accounted for by age. This is consistent with an earlier lifespan study (*n* = 388, 20–80 years), which found an age-dependent association between resting HRV and vmPFC functional connectivity, also driven by younger adults.^29^ Neural correlates of HRV might be less reliably detected in older adults due to reduced reliability of neural assessments (e.g. increased head motion) and/or reduced specificity of neural measures due to, e.g. neural dedifferentiation^102^ or early neurodegenerative processes that alter neural activity in relation to brain structure.^103^ Compared to younger adults, cognitively healthy older adults might be expected to show greater variation in neural activity in relation to cognitive (reappraisal) performance, which may be related to compensatory processes or reduced efficiency.^104^ Longitudinal trajectories of physiological, cognitive and neural measures may reveal whether compensatory processes could account for these observations.^105^ Additionally, the potential influence of dispositional emotion regulation measures on cognitive reappraisal performance^106^ in older and younger adults would be valuable to investigate in future studies.

Our findings applied only to resting HRV ECG measures, and not HRV reactivity as represented by a latent change score using PPG measures. Compared to resting HRV, HRV reactivity has been less frequently reported in relation to emotion regulation.^11,12^ Studies have shown more positive HRV change from rest during cognitive reappraisal,^107,108^ but data on the relationship between differences in HRV change and cognitive reappraisal *ability* remain unclear. In our study, using a latent change score was likely to provide a more reliable measure of HRV reactivity compared to (task minus baseline) difference scores,^109^ although other indices e.g. rate of change or time to HRV rebound via a quadratic model, or a more data-driven analysis of all PPG data (across rest and movie-watching), may better reflect central autonomic network flexibility.^13^ In addition, a more specific emotion-regulation task may have provided a better measure of HRV reactivity compared to a movie that was not specifically designed to probe emotion regulation. PPG measures, obtained whilst participants were inside an MRI scanner, have been reported to be less accurate and/or reliable versus ECG measures.^93,94^ This was supported by the higher number of arrhythmia segments found in the PPG data, a weak correlation between resting ECG and PPG measures (*r* = 0.27), and lack of an expected age-related decrease in resting HRV using PPG, but not ECG measures. The covariances between emotion regulation and either PPG or ECG were also significantly different, according to LRTs comparing model fit when the paths were freely estimated versus constrained to be equal.

### Limitations

We pre-registered a specific relationship between HRV, emotion regulation and LC integrity (in older versus younger adults) and specific variables to represent these constructs, but alternative models and indices might have fit the data better. The emotion regulation variables chosen were specific to some but not all levels of a proposed emotion processing and cardiac parasympathetic control hierarchy,^1,110^ and there is overlap of regions involved in emotion generation and regulation.^1,111^ It is possible that other regions or networks, and modeling neural compensation,^105^ could have provided a more complete understanding of the HRV–emotion regulation relationship with increasing age. Although we had aimed to include latent factors to represent HRV and emotion regulation, the main variables of interest (resting HRV and negative reappraisal) in the final models were ultimately based on single indicators, thus our findings are more vulnerable to the effects of measurement error. For example, self-reported reappraisal ratings can be influenced by (meta-) cognitive ability, and we cannot account for the possible use of other emotion regulation strategies, e.g. attentional deployment. This may partly explain why the negative reappraisal outcomes did not support a theory-driven ‘negative regulation’ latent factor^83^ and did not show an expected positive association with HRV in younger adults, in contrast to the positive regulation factor. We also averaged neural measures that were highly correlated between hemispheres in the whole sample to optimize model fit,^112^ but this may have obscured potential hemispheric differences, especially between age subgroups, in the relationship with HRV. We divided the sample into older and younger adults using a cut point of 57 years due to our theoretical framework attributing LC MRI signal intensity differences to age-related LC cell loss in older (and not younger) adults. The dichotomization of age is associated with loss of power and may have attenuated or obscured estimated effects between HRV and emotion regulation.^113^ We used chronological age, whereas markers of biological age^114,115^ may have differential effects on the heart^116^ and brain.^117^ We also did not distinguish between younger (18–35 years) and middle-aged (35–55 years) individuals, which may have obscured potential midlife-specific associations between HRV and emotion regulation.

As a component of short-term HRV is the respiration-driven increase and decrease of HR via the baroreceptor reflex and vagus nerve, known as respiratory sinus arrhythmia (RSA), differences in respiration or physical exercise can potentially bias estimation of cardiac parasympathetic control, more so for HF-HRV than RMSSD.^118,119^ Although some methodological reviews have not recommended the routine correction for respiration, due to apparent minimal effects of respiration on resting state parasympathetic HRV indices and concerns that correction for respiration may remove some of the variance associated with the common neural origin of HRV and respiration,^14^ more recent methodological guidelines recommend co-recording of respiration and reporting of respiration-adjusted analyses.^119^ As we did not have co-recorded respiration data, it is possible that age-related differences in respiratory efficiency and function may have confounded the observed relationship between HRV and reappraisal performance.

Our fMRI denoising strategy may have contributed to the low loadings of regional resting state functional connectivity measures to the emotion regulation latent factor, as a higher number of nuisance signal regressors can reduce associations between HRV and functional connectivity.^120^ Other approaches to measuring resting state functional connectivity, such as eigenvector centrality mapping,^29^ may have better reflected central autonomic network pathways. The small size of the LC also likely limited our ability to capture LC-ROI functional connectivity measures, especially with relatively low fMRI resolution and spatial smoothing.

Technical limitations may have influenced the reliability of LC MRI signal intensity values, which were obtained from an earlier study,^36^ including relatively low resolution of MT-weighted images, and the statistical correction for different repetition times (TR) (either 30 ms or 50 ms) used in the structural imaging protocol may not have fully accounted for the effects of the differences. There was no correlation between the LC MRI signal intensity and LC functional gradient measures, so the differential associations with HRV need further study. We may have been limited by insufficient power to find an LC effect on the HRV–emotion regulation relationship, particularly in a non-clinical sample. The nature and degree of missing data may also have limited the reliability of the findings on the relationship between HRV and reappraisal outcomes, as Cam-CAN participants were invited to attend either the session that included the ERRT or a different session that did not include the ERRT, leading to only *n* = 314 completing the ERRT, thus only *n* = 130 older adults’ and *n* = 160 younger adults’ reappraisal task scores were available. We did not control for multiple comparisons, and the cross-sectional nature and sample size of our data meant that we were unlikely to have sufficient power or to have been able to establish the hypothesized directionality of the HRV–emotion regulation relationship via pre-registered moderation analyses. Future longitudinal studies are needed to investigate potential causal or compensatory trajectories of HRV–emotion regulation in older adults,^17^ as well as potential LC moderation effects.

We only included the Cattell Fair score as a measure of fluid intelligence and did not include other measures of specific cognitive domains, e.g. executive function, in the models, which may have been informative. Potential factors influencing the HRV–emotion regulation relationship, such as body mass index or lateralization effects (e.g. handedness), also need further study so that any observed statistical effects can be interpreted within a theoretical framework. We did not have data on other medications, e.g. antidepressants with noradrenergic effects, which can potentially influence HRV and emotion regulation. Although the study sample was population-based, it was not population-representative, with cognitive performance thresholds required for participation, and participation was related to deprivation level and age.^121^ Our findings may not generalize to more diverse samples, as ethnicity can influence HRV^122,123^ and emotion regulation,^124^ but study participants had predominantly White European ancestry.

## Conclusion

Contrary to the prevailing hypothesis that higher HRV is associated with better emotion regulation, our study found that resting HRV was inversely related to emotion regulation performance measures in older adults, and this relationship significantly differed from that seen in younger adults. Our findings reveal knowledge gaps in autonomic system changes in aging and neurodegenerative disorders and their implications for emotional health. It will be important to try to replicate these findings and conduct studies that incorporate longitudinal designs, diverse clinical populations including Alzheimer’s disease, and more granular measures of emotion regulation strategies.

## Supplementary Material

fcaf395_Supplementary_Data

## Data Availability

Data collection and sharing for this project was provided by the Cambridge Centre for Ageing and Neuroscience (Cam-CAN). The Cam-CAN is an open-access data set available at https://camcan-archive.mrc-cbu.cam.ac.uk/dataaccess/.^125^ Our hypotheses and analysis plan were pre-registered after data had been collected but before analyses were undertaken (see https://aspredicted.org/blind.php?x=LXY_VCQ). The R code that supports the SEM analyses within this paper is available at https://github.com/k-y-liu/HRV-ER_CamCANstudy. The LC ROI mask used in the study (available from https://osf.io/r2bwk/)^80^ was obtained from a previous study in Cam-CAN participants.^36^

## Appendix

Cam-CAN project principal personnel: Richard N. Henson, Lorraine K. Tyler, Kamen A. Tsvetanov, Carol Brayne, Edward T. Bullmore, Andrew C. Calder, Rhodri Cusack, Tim Dalgleish, John Duncan, Fiona E. Matthews, William D. Marslen-Wilson, James B. Rowe, Meredith A. Shafto, Marta Correia; Research Associates: Karen Campbell, Teresa Cheung, Simon Davis, Linda Geerligs, Rogier Kievit, Anna McCarrey, Abdur Mustafa, Darren Price, David Samu, Jason R. Taylor, Matthias Treder, Janna van Belle, Nitin Williams, Daniel Mitchell, Simon Fisher, Else Eising, Ethan Knights, Adam Attaheri, Dace Apsvalka, Maite Crespo-Garcia; Research Assistants: Lauren Bates, Tina Emery, Sharon Erzinçlioğlu, Andrew Gadie, Sofia Gerbase, Stanimira Georgieva, Claire Hanley, Beth Parkin, David Troy, Ina Demetriou, Will Duckett; Affiliated Personnel: Tibor Auer, Lu Gao, Emma Green, Rafael Henriques; Research Interviewers: Jodie Allen, Gillian Amery, Liana Amunts, Anne Barcroft, Amanda Castle, Cheryl Dias, Jonathan Dowrick, Melissa Fair, Hayley Fisher, Anna Goulding, Adarsh Grewal, Geoff Hale, Andrew Hilton, Frances Johnson, Patricia Johnston, Thea Kavanagh-Williamson, Magdalena Kwasniewska, Alison McMinn, Kim Norman, Jessica Penrose, Fiona Roby, Diane Rowland, John Sargeant, Maggie Squire, Beth Stevens, Aldabra Stoddart, Cheryl Stone, Tracy Thompson, Ozlem Yazlik; and administrative staff: Dan Barnes, Marie Dixon, Jaya Hillman, Joanne Mitchell, Laura Villis.
