# Supplementary material for: Different association patterns of emotion regulation and heart rate variability in older and younger adults
Source: Brain Commun. 2025 Oct 10;7(5):fcaf395. doi: 10.1093/braincomms/fcaf395 (PMC12569767; doi:10.1093/braincomms/fcaf395)
Supplement: fcaf395_Supplementary_Data [file fcaf395_supplementary_data.docx]

**Supplemental Information**

[**Supplementary Table 1: Cam-CAN MRI acquisition details** 1](#_Toc210809229)

[**Supplementary Methods 1: HRV measurement model** 2](#_Toc210809230)

[**Supplementary Figure 1: Pre-registered HRV latent factor measurement model.** 3](#_Toc210809231)

[**Supplementary Figure 2: Latent change score measurement model for HRV reactivity (via PPG)** 4](#_Toc210809232)

[**Supplementary Methods 2: Emotion regulation measurement models** 5](#_Toc210809233)

[**Supplementary Figure 3: Emotion regulation latent factor measurement models** 6](#_Toc210809234)

[**Supplementary Table 2: Additional descriptive statistics for HRV, primary emotion regulation and LC variables, and demographic covariates** 8](#_Toc210809235)

[**Supplementary Table 3: Descriptive statistics for secondary emotion regulation variables** 10](#_Toc210809236)

[**Supplementary Table 4: Correlation matrix of pairwise (Pearson’s r) correlations between analyzed variables (whole sample).** 11](#_Toc210809237)

[**Supplementary Table 5: Correlation matrix of pairwise (Pearson’s r) correlations between analyzed variables (younger group <57 years).** 12](#_Toc210809238)

[**Supplementary Table 6: Correlation matrix of pairwise (Pearson’s r) correlations between analyzed variables (older group ≥57 years).** 13](#_Toc210809239)

[**Supplementary Table 7: Standardized covariance/regression estimates and fit indices for the correlational models.** 14](#_Toc210809240)

[**Supplementary Figure 4: Correlational multigroup SEM model for resting HRV and four latent emotionality factors** 18](#_Toc210809241)

[**Supplementary Results 1: Analytical power for moderation analysis in cross-sectional data** 20](#_Toc210809242)

### **Supplementary Table 1: Cam-CAN MRI acquisition details**

| Scan type | Sequence | TR (ms) | TE (ms) | Flip angle (^o^) | FOV (mm) | Voxel size (mm) | Other |
| --- | --- | --- | --- | --- | --- | --- | --- |
| T1-weighted | MPRAGE | 2250 | 2.99 | 9 | 256 × 240 × 19 | 1x1x1 | GRAPPA: 2; TI: 900 ms |
| Diffusion-weighted |  |  |  |  |  |  |  |
| b = 1000 | Twice-refocused SE | 9100 | 104 |  | 192 × 192 | 2 × 2 × 2 | directions: 30; slices: 66 (axial); averages: 1 |
| b = 2000 | Twice-refocused SE | 9100 | 104 |  | 192 × 192 | 2 × 2 × 2 | directions: 30; slices: 66 (axial); averages: 1 |
| b = 0 | Twice-refocused SE | 9100 | 104 |  | 192 × 192 | 2 × 2 × 2 | slices: 66 (axial); images: 3 |
| Magnetisation transfer |  |  |  |  |  |  |  |
| Baseline | MT-prepared SPGR | 30^a^ | 5 |  | 192 × 192 | 1.5 × 1.5 × 1.5 | bandwidth: 190 Hz/px |
| MT | MT-prepared SPGR | 30^a^ | 5 |  | 192 × 192 | 1.5 × 1.5 × 1.5 | bandwidth: 190 Hz/px; RF pulse applied^b^ |
| Resting state | EPI | 1970 | 30 | 78 | 192 × 192 | 3 × 3 × 4.44 | volumes: 261; slices: 32; slice thickness: 3.7mm; gap: 20%; order: descending |

Information obtained from Taylor. et al ^1^.

Abbreviations: TR = repetition time; TE = echo time; TI = inversion time; FOV = field of view; MPRAGE = magnetisation prepared gradient echo; SE = spin echo; MT = magnetisation transfer; SPGR = spoiled gradient; EPI = T2*-weighted gradient echo echo planar image.

^a^ TR = 50 used if SAR exceeded limits.

^b^ RF pulse: Gaussian RF pulse, 1950Hz (bandwidth = 375Hz, flip angle = 500°, duration = 9984 μs).

### **Supplementary Methods 1: HRV measurement model**

Initial confirmatory factor analysis (CFA) showed that, although the pre-specified HRV latent factor model (Supplementary Figure 1) displayed adequate fit measures (CFI=1, RMSEA=0, SRMR=0.017), the individual standardized factor loadings were low (<0.34), and at least one eigenvalue was negative. This was likely related to the high collinearity between pairs of HF and RMSSD measures (*r* > 0.9), as shown in Supplementary Tables 4-6. Thus other model structures were explored; a bifactor model (with ECG and PPG latent ‘modality’ factors) showed similar limitations, and a second order model (with latent rest and reactivity factors) also offered no improvement as the two factors did not correlate.

To manage the high collinearity between pairs of HF and RMSSD measures, we calculated average HRV scores resulting in one mean ECG (resting) and two mean PPG (resting and movie-watching) variables to represent vagally-mediated HRV. Initially, we explored a latent HRV factor model comprising these three averaged variables, where the PPG residuals were allowed to covary. Although this showed acceptable fit (CFI 0.995, RMSEA 0.068, SRMR 0.013), subsequent multigroup analyses showed a negative variance in the latent HRV factor in older adults. Closer inspection revealed differential relationships with age between ECG and PPG measures and, given also the poor correlation between resting ECG and PPG (*r =* 0.27) and the likely higher accuracy and reliability of ECG measures (especially compared to fingertip PPG ^2,3^), we decided to distinguish between these two modalities in separate models. We therefore investigated (and compared) resting HRV (i.e., the averaged HF/RMSSD measure using resting ECG), and HRV reactivity (i.e., ΔHRV in a latent change score model ^4^ using PPG obtained during rest and whilst watching a compelling and unfamiliar movie (Supplementary Figure 2), separately in the correlational models.

### **Supplementary Figure 1: Pre-registered HRV latent factor measurement model.**


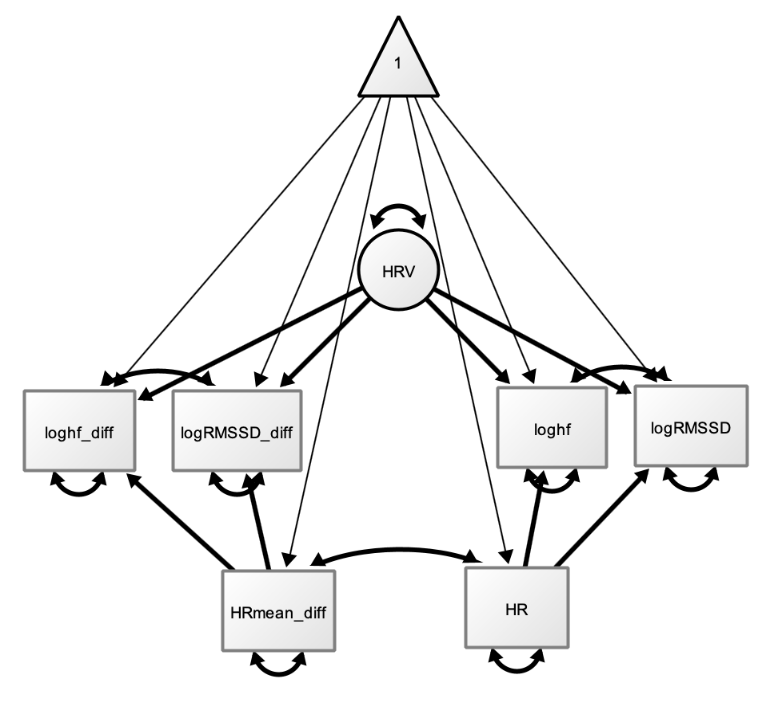


Confirmatory factor analysis of the pre-registered HRV latent factor measurement model showed acceptable fit measures (CFI=1, RMSEA=0, SRMR=0.017), but individual standardized factor loadings were low (<0.34). Observed variables were two HRV reactivity (PPG) measures (*loghf_diff* and *log_RMSSD_diff*) and two resting HRV (ECG) measures (*loghf* and *logRMSSD*), adjusted for heart rate (*HRmean_diff* and *HR*). Residuals of pairs of resting (ECG), reactivity (PPG), and HR variables were allowed to covary. The constant is represented by a triangle, the latent factor is represented by a circle and observed variables are represented by rectangles. One-headed arrows indicate regressions, double-headed arrows indicate (co)variances. Number of observations used: N=618 (5 missing patterns).

Abbreviations: CFI=comparative fit index; ECG=electrocardiogram; HR=heart rate; HRV=heart rate variability; PPG= photoplethysmography; RMSEA=root-mean-square error of approximation; SRMR= standardized root mean residual.

### **Supplementary Figure 2: Latent change score measurement model for HRV reactivity (via PPG)**

*
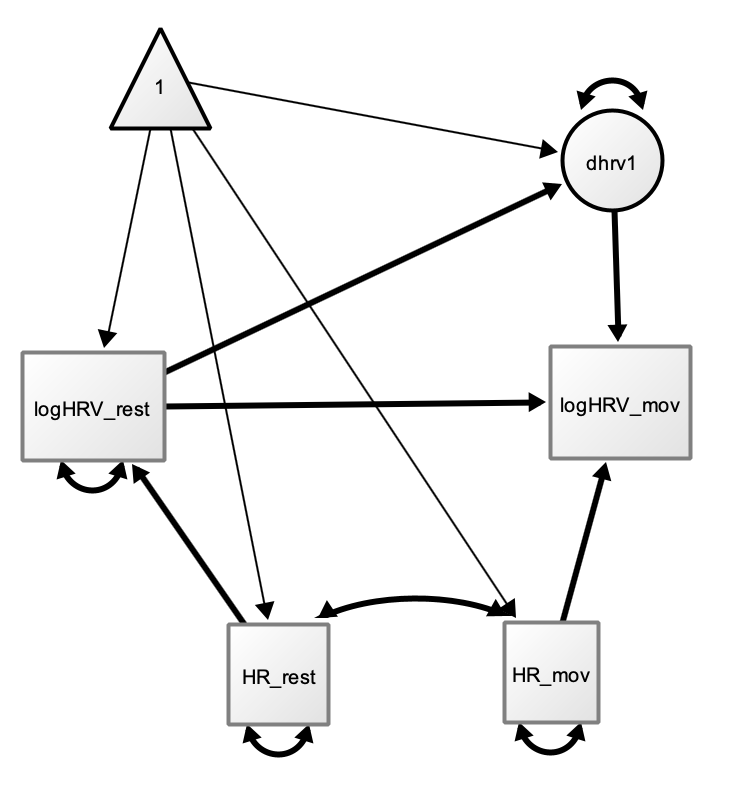
*

Confirmatory factor analysis of the HRV reactivity latent change score measurement model showed acceptable fit measures (CFI 0.996, RMSEA 0.064, SRMR 0.011). A latent change score factor (*dhrv1*) representing HRV reactivity was modelled using HRV (averaged HF/RMSSD) measures obtained using photoplethysmography (PPG) during rest (*logHRV_rest)* and movie-watching (*logHRV_mov)*, and adjusted for heart rate (*HR_rest and HR_mov*). The constant is represented by a triangle, the latent factor is represented by a circle and observed variables are represented by squares/rectangles. One-headed arrows indicate regressions, double-headed arrows indicate (co)variances. Number of observations used: N=482 (2 missing patterns).

Abbreviations: CFI=comparative fit index; HF=high frequency HRV; HR=heart rate; HRV=heart rate variability; PPG= photoplethysmography; RMSEA=root-mean-square error of approximation; RMSSD= root mean square of successive differences in normal-to-normal intervals; SRMR= standardized root mean residual.

### **Supplementary Methods 2: Emotion regulation measurement models**

To simplify the model and optimize model fit, we averaged neural measures that showed high correlations (>0.7) between hemispheres and between anterior and posterior insula volumes. Confirmatory factor analysis (CFA) of the pre-registered primary emotion regulation latent factor, where residuals of modality-specific variables were allowed to covary, showed good fit (CFI 1, RMSEA 0, SRMR 0.009) (Supplementary Figure 3A). Model fit remained good after pre-registered secondary measures were added (CFI 0.991, RMSEA 0.025, SRMR 0.028) (Supplementary Figure 3B). The resting state functional connectivity variables and the Negative Reappraisal task score showed low (<0.1) standardized factor loadings; however, only removal of the latter improved model fit (CFI=0.994, RMSEA =0.022, SRMR=0.022). This supported a distinction between ‘neural’ and ‘psychological’ emotion regulation capacity, which could plausibly differentially relate to HRV with increasing age, e.g., older adults generally show age-related reduction in cortical structural integrity, but also report better emotional wellbeing ^5^. Thus, we decided to analyze these constructs separately in the correlational model (Figure 2).

### **Supplementary Figure 3: Emotion regulation latent factor measurement models**


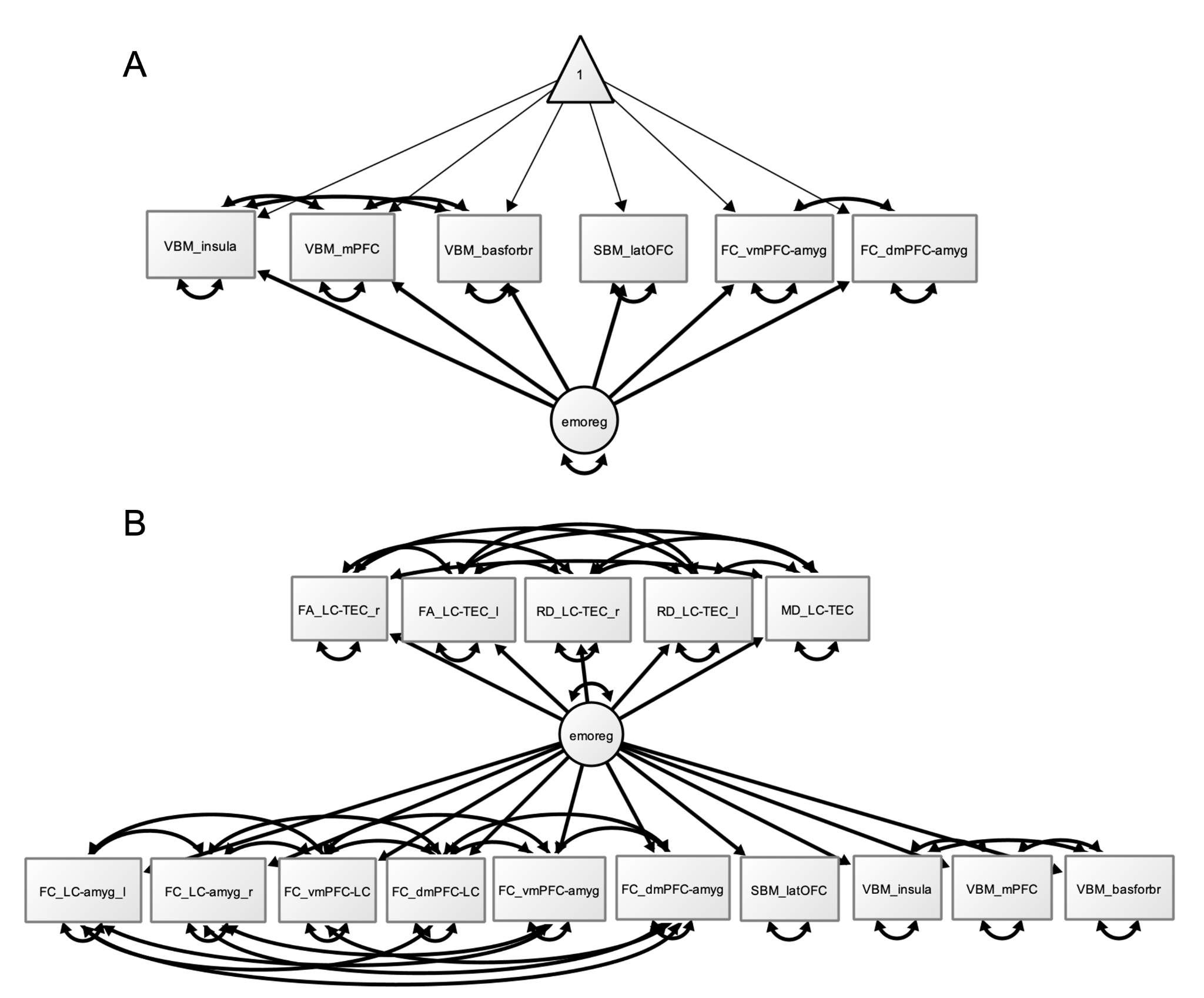


Confirmatory factor analysis of the emotion regulation latent factor (*emoreg)* measurement model, comprising primary measures (**A**) and additional secondary measures (**B**) showed good fit (Model A: CFI 1, RMSEA 0, SRMR 0.009; Model B: CFI 0.991, RMSEA 0.025, SRMR 0.028). Model A included mean total intracranial volume-adjusted insula (*VBM_insula)*, medial prefrontal cortex (PFC) (*VBM_mPFC)* and basal forebrain (*VBM_basforbr)* volumes, mean lateral orbitofrontal cortical thickness (*SBM_latOFC)*, and mean ventromedial (vm)PFC-amygdala (*FC_vmPFC-amyg)* and dorsomedial (dm)PFC-amygdala (*FC_dmPFC-amyg*) resting state functional connectivity measures. Model B additionally included locus coeruleus (LC)-transentorhinal cortex pathway diffusion tensor metrics, including bilateral fractional anisotropy (*FA_LC-TEC_r, FA_LC-TEC_l*), radial diffusivity (*RD_LC-TEC_r, RD_LC-TEC_l*), and mean diffusivity (*MD_LC-TEC)*, additional resting state functional connectivity measures, including mean vmPFC-LC (*FC_vmPFC-LC)*, dmPFC-LC (*FC_dmPFC-LC)*, and LC-bilateral amygdala (*FC-LC-amyg_l and FC_LC-amyg_r)* connectivity, and Negative Reappraisal task score (not shown). The constant is represented by a triangle (not shown in (B) to aid clarity), the latent factor is represented by a circle and observed variables are represented by squares/rectangles. One-headed arrows indicate regressions, double-headed arrows indicate (co)variances. Number of observations used: N=653 (2 missing patterns).

Abbreviations:

CFI=comparative fit index; RMSEA=root-mean-square error of approximation; SRMR= standardized root mean residual.

### **Supplementary Table 2: Additional descriptive statistics for HRV, primary emotion regulation and LC variables, and demographic covariates**

| Variable | Scaled mean (SD)^f^ | | Kurtosis | | Skewness | |
| --- | --- | --- | --- | --- | --- | --- |
|  | Younger group | Older group | Younger group | Older group | Younger group | Older group |
| Age | 3.40 (1.10) | 6.86 (0.90) | 1.99 | 1.88 | -0.18 | 0.02 |
| % Female |  |  |  |  |  |  |
| Age when completed full time education | 5.37 (1.60) | 4.58 (2.31) | 3.33 | 10.3 | 0.26 | 1.47 |
| Cattell fair total score | 6.10 (1.36) | 3.73 (1.86) | 2.85 | 2.62 | -0.60 | -0.08 |
| Cardiovascular health status score^a^ | 4.3 (1.1) | 5.9 (2.4) | 18.44 | 3.86 | 3.64 | 1.17 |
| % Beta-blocker drug present^b^ |  |  |  |  |  |  |
| Negative Reappraisal score | 5.01 (1.96) | 4.99 (2.05) | 3.22 | 7.19 | 0.17 | 0.28 |
| logHRV^c^ (resting ECG) | 5.33 (1.49) | 4.52 (2.45) | 3.40 | 2.51 | 0.10 | 0.20 |
| logHRV^c^ (resting PPG) | 5.04 (2.02) | 4.96 (1.94) | 6.29 | 4.65 | -1.21 | -0.83 |
| logHRV^c^ (movie PPG) | 4.99 (2.07) | 5.01 (1.88) | 4.08 | 5.34 | -0.55 | -0.76 |
| VBM mPFC^d^ | 5.94 (1.58) | 3.93 (1.59) | 3.44 | 3.19 | 0.32 | -0.02 |
| VBM basal forebrain^d^ | 5.91 (1.63) | 3.97 (1.67) | 3.00 | 3.32 | 0.28 | 0.24 |
| VBM insula^d^ | 5.85 (1.64) | 4.03 (1.54) | 2.96 | 3.07 | 0.32 | 0.11 |
| SBM lateral OFC | 5.62 (1.65) | 4.30 (1.89) | 3.07 | 3.12 | 0.06 | -0.06 |
| Mean LC CR^e^ | 4.57 (1.77) | 5.51 (2.14) | 3.66 | 3.55 | 0.21 | 0.07 |

Number of observations, scaled values, skewness, and kurtosis are shown for descriptive purposes.

^a^ Cardiovascular health status score was the total number of conditions present out of diabetes, stroke, hypertension, high cholesterol, myocardial infarction.

^b^ Beta-blocker drug status was a binary measure (absent/present).

^c^ LogHRV values were derived by averaging logHF and logRMSSD scores, which were highly correlated (r>0.9).

^d^ Brain volumes were adjusted for (divided by) total intracranial volume.

^e^ LC MRI signal intensities were normalized to a pontine reference region.

^f^ All continuous variables were scaled to have mean=5 and SD=2 across the whole sample to facilitate model estimation.

Abbreviations: HRV=heart rate variability, VBM=voxel-based morphometry, mPFC=medial prefrontal cortex, SBM=surface-based morphometry, OFC=orbitofrontal, LC CR=locus coeruleus contrast ratio.

### **Supplementary Table 3: Descriptive statistics for secondary emotion regulation variables**

| Variable | No. of observations (% of total) | | Scaled mean (SD) | | Kurtosis | | Skewness | |
| --- | --- | --- | --- | --- | --- | --- | --- | --- |
|  | Younger group | Older group | Younger group | Older group | Younger group | Older group | Younger group | Older group |
| FC dmPFC-amygdala | 346 (95.1) | 306 (97.5) | 5.05 (1.74) | 4.95 (1.97) | 3.40 | 3.22 | -0.32 | -0.20 |
| FC vmPFC-amygdala | 346 (95.1) | 306 (97.5) | 4.95 (1.77) | 5.05 (1.92) | 3.07 | 3.42 | 0.13 | 0.31 |
| FC dmPFC-LC | 346 (95.1) | 306 (97.5) | 5.07 (1.96) | 4.93 (2.05) | 3.08 | 4.03 | 0.11 | 0.16 |
| FC vmPFC-LC | 346 (95.1) | 306 (97.5) | 5.12 (2.02) | 4.86 (1.97) | 3.07 | 3.27 | -0.11 | -0.09 |
| FC LC-amygdala_l | 346 (95.1) | 306 (97.5) | 5.11 (1.90) | 4.88 (2.11) | 3.07 | 3.16 | 0.12 | 0.29 |
| FC LC-amygdala_r | 346 (95.1) | 306 (97.5) | 5.07 (1.93) | 4.92 (2.07) | 3.64 | 2.99 | 0.04 | -0.02 |
| MD LC-TEC | 337 (92.6) | 304 (96.8) | 4.15 (0.98) | 5.94 (2.24) | 16.5 | 4.22 | 1.51 | 1.09 |
| FA LC-TEC_l | 337 (92.6) | 304 (96.8) | 5.30 (1.81) | 4.67 (2.15) | 65.5 | 35.44 | -5.53 | -4.76 |
| FA LC-TEC_r | 337 (92.6) | 304 (96.8) | 5.79 (1.79) | 4.12 (1.85) | 50.3 | 21.1 | 4.74 | -2.46 |
| RD LC-TEC_l | 337 (92.6) | 304 (96.8) | 4.24 (1.08) | 5.85 (2.41) | 20.19 | 5.57 | 2.78 | 1.51 |
| RD LC-TEC_r | 337 (92.6) | 304 (96.8) | 4.02 (1.28) | 6.09 (2.09) | 9.73 | 4.88 | 0.51 | 0.40 |

Data are shown for younger (N=364) and older (N=314) groups. All continuous variables were scaled to have mean=5 and SD=2 across the whole sample to optimize model fit. Abbreviations: FC= functional connectivity, vmPFC=ventromedial prefrontal cortex, dmPFC=dorsomedial prefrontal cortex, LC=locus coeruleus, MD= mean diffusivity, TEC=transentorhinal cortex, FA=fractional anisotropy, RD=radial diffusivity, r/l=right/left.

### **Supplementary Table 4: Correlation matrix of pairwise (Pearson’s r) correlations between analyzed variables (whole sample).**

Only significant (p<0.05) correlations are shown in bold.

|  | 1 | 2 | 3 | 4 | 5 | 6 | 7 | 8 | 9 | 10 | 11 | 12 | 13 | 14 | 15 | 16 | 17 | 18 | 19 | 20 | 21 | 22 | 23 | 24 |
| --- | --- | --- | --- | --- | --- | --- | --- | --- | --- | --- | --- | --- | --- | --- | --- | --- | --- | --- | --- | --- | --- | --- | --- | --- |
| 1.logRMSSD_restECG | 1.00 |  |  |  |  |  |  |  |  |  |  |  |  |  |  |  |  |  |  |  |  |  |  |  |
| 2.logHF_restECG | **0.96** | 1.00 |  |  |  |  |  |  |  |  |  |  |  |  |  |  |  |  |  |  |  |  |  |  |
| 3.logHF_movPPG | **0.28** | **0.26** | 1.00 |  |  |  |  |  |  |  |  |  |  |  |  |  |  |  |  |  |  |  |  |  |
| 4.logHF_restPPG | **0.30** | **0.27** | **0.74** | 1.00 |  |  |  |  |  |  |  |  |  |  |  |  |  |  |  |  |  |  |  |  |
| 5.logRMSSD_movPPG | **0.26** | **0.25** | **0.96** | **0.72** | 1.00 |  |  |  |  |  |  |  |  |  |  |  |  |  |  |  |  |  |  |  |
| 6.logRMSSD_restPPG | **0.26** | **0.24** | **0.73** | **0.96** | **0.75** | 1.00 |  |  |  |  |  |  |  |  |  |  |  |  |  |  |  |  |  |  |
| 7.VBM basal forebrain |  |  |  |  |  |  | 1.00 |  |  |  |  |  |  |  |  |  |  |  |  |  |  |  |  |  |
| 8.VBM mPFC |  | **0.22** |  |  |  |  | **0.60** | 1.00 |  |  |  |  |  |  |  |  |  |  |  |  |  |  |  |  |
| 9.VBM Insula |  | **0.23** |  |  |  |  | **0.70** | **0.63** | 1.00 |  |  |  |  |  |  |  |  |  |  |  |  |  |  |  |
| 10.FC dmPFC-amyg |  |  |  |  |  |  |  |  |  | 1.00 |  |  |  |  |  |  |  |  |  |  |  |  |  |  |
| 11.FC vmPFC-amyg |  |  |  |  |  |  |  |  |  | **0.51** | 1.00 |  |  |  |  |  |  |  |  |  |  |  |  |  |
| 12.SBM lateral OFC | **0.21** | **0.26** |  |  |  |  | **0.43** | **0.37** | **0.42** |  |  | 1.00 |  |  |  |  |  |  |  |  |  |  |  |  |
| 13.FC LC-amyg_l |  |  |  |  |  |  |  |  |  |  |  |  | 1.00 |  |  |  |  |  |  |  |  |  |  |  |
| 14.FC LC-amyg_r |  |  |  |  |  |  |  |  |  |  |  |  | **0.67** | 1.00 |  |  |  |  |  |  |  |  |  |  |
| 15.FC dmPFC-LC |  |  |  |  |  |  |  |  |  | **0.15** |  |  |  |  | 1.00 |  |  |  |  |  |  |  |  |  |
| 16.FC vmPFC-LC |  |  |  |  |  |  |  |  |  |  |  |  | **0.16** |  | **0.52** | 1.00 |  |  |  |  |  |  |  |  |
| 17.FA LC-TEC_l |  |  |  |  |  |  |  |  |  |  |  |  |  |  |  |  | 1.00 |  |  |  |  |  |  |  |
| 18.FA LC-TEC_r |  | **0.20** |  |  |  |  | **0.40** | **0.39** | **0.33** |  |  | **0.25** |  |  |  |  | **0.43** | 1.00 |  |  |  |  |  |  |
| 19.MD LC-TEC |  |  |  |  |  |  | **-0.40** | **-0.42** | **-0.34** |  |  | **-0.32** |  |  |  |  | **-0.18** | **-0.37** | 1.00 |  |  |  |  |  |
| 20.RD LC-TEC_l |  |  |  |  |  |  | **-0.33** | **-0.37** | **-0.26** |  |  | **-0.30** |  |  |  |  | **-0.18** | **-0.32** | **0.85** | 1.00 |  |  |  |  |
| 21.RD LC-TEC_r |  |  |  |  |  |  | **-0.51** | **-0.45** | **-0.45** |  |  | **-0.28** |  |  |  |  | **-0.20** | **-0.44** | **0.52** | **0.46** | 1.00 |  |  |  |
| 22.Neg_reappraisal |  |  |  |  |  |  |  |  |  |  |  |  |  |  |  |  |  |  |  |  |  | 1.00 |  |  |
| 23.mean LCCR |  |  |  |  |  |  | **-0.25** | **-0.20** | **-0.22** |  |  |  |  |  |  |  |  | **-0.17** |  |  |  |  | 1.00 |  |
| 24.age | **-0.18** | **-0.28** |  |  |  |  | **-0.63** | **-0.65** | **-0.60** |  |  | **-0.44** |  |  |  |  |  | **-0.45** | **0.54** | **0.48** | **0.58** |  | **0.30** | 1.00 |

### **Supplementary Table 5: Correlation matrix of pairwise (Pearson’s r) correlations between analyzed variables (younger group <57 years).**

Only significant (p<0.05) correlations are shown in bold.

|  | 1 | 2 | 3 | 4 | 5 | 6 | 7 | 8 | 9 | 10 | 11 | 12 | 13 | 14 | 15 | 16 | 17 | 18 | 19 | 20 | 21 | 22 | 23 | 24 |
| --- | --- | --- | --- | --- | --- | --- | --- | --- | --- | --- | --- | --- | --- | --- | --- | --- | --- | --- | --- | --- | --- | --- | --- | --- |
| 1.logRMSSD_restECG | 1.00 |  |  |  |  |  |  |  |  |  |  |  |  |  |  |  |  |  |  |  |  |  |  |  |
| 2.logHF_restECG | **0.93** | 1.00 |  |  |  |  |  |  |  |  |  |  |  |  |  |  |  |  |  |  |  |  |  |  |
| 3.logHF_movPPG | **0.36** | **0.31** | 1.00 |  |  |  |  |  |  |  |  |  |  |  |  |  |  |  |  |  |  |  |  |  |
| 4.logHF_restPPG | **0.42** | **0.34** | **0.72** | 1.00 |  |  |  |  |  |  |  |  |  |  |  |  |  |  |  |  |  |  |  |  |
| 5.logRMSSD_movPPG | **0.35** | **0.30** | **0.96** | **0.73** | 1.00 |  |  |  |  |  |  |  |  |  |  |  |  |  |  |  |  |  |  |  |
| 6.logRMSSD_restPPG | **0.38** | **0.31** | **0.71** | **0.97** | **0.75** | 1.00 |  |  |  |  |  |  |  |  |  |  |  |  |  |  |  |  |  |  |
| 7.VBM basal forebrain |  |  |  |  |  |  | 1.00 |  |  |  |  |  |  |  |  |  |  |  |  |  |  |  |  |  |
| 8.VBM mPFC |  |  |  |  |  |  | **0.42** | 1.00 |  |  |  |  |  |  |  |  |  |  |  |  |  |  |  |  |
| 9.VBM Insula |  | **0.26** |  |  |  |  | **0.68** | **0.51** | 1.00 |  |  |  |  |  |  |  |  |  |  |  |  |  |  |  |
| 10.FC dmPFC-amyg |  |  |  |  |  |  |  |  |  | 1.00 |  |  |  |  |  |  |  |  |  |  |  |  |  |  |
| 11.FC vmPFC-amyg |  |  |  |  |  |  |  |  |  | **0.46** | 1.00 |  |  |  |  |  |  |  |  |  |  |  |  |  |
| 12.SBM lateral OFC |  |  |  |  |  |  | **0.37** |  | **0.34** |  |  | 1.00 |  |  |  |  |  |  |  |  |  |  |  |  |
| 13.FC LC-amyg_l |  |  |  |  |  |  |  |  |  |  |  |  | 1.00 |  |  |  |  |  |  |  |  |  |  |  |
| 14.FC LC-amyg_r |  |  |  |  |  |  |  |  |  |  |  |  | **0.73** | 1.00 |  |  |  |  |  |  |  |  |  |  |
| 15.FC dmPFC-LC |  |  |  |  |  |  |  |  |  |  |  |  | **0.21** |  | 1.00 |  |  |  |  |  |  |  |  |  |
| 16.FC vmPFC-LC |  |  |  |  |  |  |  |  |  |  |  |  |  |  | **0.59** | 1.00 |  |  |  |  |  |  |  |  |
| 17.FA LC-TEC_l |  |  |  |  |  |  |  |  |  |  |  |  |  |  |  |  | 1.00 |  |  |  |  |  |  |  |
| 18.FA LC-TEC_r |  |  |  |  |  |  | **0.20** |  |  |  |  |  |  |  |  |  | **0.34** | 1.00 |  |  |  |  |  |  |
| 19.MD LC-TEC |  |  |  |  |  |  |  |  |  |  |  |  |  |  |  |  |  | **-0.32** | 1.00 |  |  |  |  |  |
| 20.RD LC-TEC_l |  |  |  |  |  |  |  |  |  |  |  |  |  |  |  |  |  | **-0.25** | **0.81** | 1.00 |  |  |  |  |
| 21.RD LC-TEC_r |  |  |  |  |  |  | **-0.33** | **-0.23** | **-0.24** |  |  |  |  |  |  |  | **-0.21** | **-0.46** | **0.42** | **0.33** | 1.00 |  |  |  |
| 22.Neg_reappraisal |  |  |  |  |  |  |  |  |  |  |  |  |  |  |  |  |  |  |  |  |  | 1.00 |  |  |
| 23.mean LCCR |  |  |  |  |  |  | **-0.25** | **-0.24** | **-0.22** |  |  |  |  |  |  |  |  |  |  |  |  |  | 1.00 |  |
| 24.age | **-0.30** | **-0.41** |  |  |  |  | **-0.44** | **-0.46** | **-0.49** |  |  | **-0.33** |  |  |  |  |  |  |  |  | **0.22** |  | **0.43** | 1.00 |

### **Supplementary Table 6: Correlation matrix of pairwise (Pearson’s r) correlations between analyzed variables (older group ≥57 years).**

Only significant (p<0.05) correlations are shown in bold.

|  | 1 | 2 | 3 | 4 | 5 | 6 | 7 | 8 | 9 | 10 | 11 | 12 | 13 | 14 | 15 | 16 | 17 | 18 | 19 | 20 | 21 | 22 | 23 | 24 |
| --- | --- | --- | --- | --- | --- | --- | --- | --- | --- | --- | --- | --- | --- | --- | --- | --- | --- | --- | --- | --- | --- | --- | --- | --- |
| 1.logRMSSD_restECG | 1.00 |  |  |  |  |  |  |  |  |  |  |  |  |  |  |  |  |  |  |  |  |  |  |  |
| 2.logHF_restECG | **0.98** | 1.00 |  |  |  |  |  |  |  |  |  |  |  |  |  |  |  |  |  |  |  |  |  |  |
| 3.logHF_movPPG |  |  | 1.00 |  |  |  |  |  |  |  |  |  |  |  |  |  |  |  |  |  |  |  |  |  |
| 4.logHF_restPPG |  |  | **0.76** | 1.00 |  |  |  |  |  |  |  |  |  |  |  |  |  |  |  |  |  |  |  |  |
| 5.logRMSSD_movPPG |  |  | **0.96** | **0.73** | 1.00 |  |  |  |  |  |  |  |  |  |  |  |  |  |  |  |  |  |  |  |
| 6.logRMSSD_restPPG |  |  | **0.75** | **0.96** | **0.76** | 1.00 |  |  |  |  |  |  |  |  |  |  |  |  |  |  |  |  |  |  |
| 7.VBM basal forebrain |  |  |  |  |  |  | 1.00 |  |  |  |  |  |  |  |  |  |  |  |  |  |  |  |  |  |
| 8.VBM mPFC |  |  |  |  |  |  | **0.49** | 1.00 |  |  |  |  |  |  |  |  |  |  |  |  |  |  |  |  |
| 9.VBM Insula |  |  |  |  |  |  | **0.52** | **0.47** | 1.00 |  |  |  |  |  |  |  |  |  |  |  |  |  |  |  |
| 10.FC dmPFC-amyg |  |  |  |  |  |  |  |  |  | 1.00 |  |  |  |  |  |  |  |  |  |  |  |  |  |  |
| 11.FC vmPFC-amyg |  |  |  |  |  |  |  |  |  | **0.55** | 1.00 |  |  |  |  |  |  |  |  |  |  |  |  |  |
| 12.SBM lateral OFC |  |  |  |  |  |  | **0.27** | **0.27** | **0.25** |  |  | 1.00 |  |  |  |  |  |  |  |  |  |  |  |  |
| 13.FC LC-amyg_l |  |  |  |  |  |  |  |  |  |  |  |  | 1.00 |  |  |  |  |  |  |  |  |  |  |  |
| 14.FC LC-amyg_r |  |  |  |  |  |  |  |  |  |  |  |  | **0.61** | 1.00 |  |  |  |  |  |  |  |  |  |  |
| 15.FC dmPFC-LC |  |  |  |  |  |  |  |  |  |  |  |  |  |  | 1.00 |  |  |  |  |  |  |  |  |  |
| 16.FC vmPFC-LC |  |  |  |  |  |  |  |  |  |  |  |  |  |  | **0.44** | 1.00 |  |  |  |  |  |  |  |  |
| 17.FA LC-TEC_l |  |  |  |  |  |  |  |  |  |  |  |  |  |  |  |  | 1.00 |  |  |  |  |  |  |  |
| 18.FA LC-TEC_r |  |  |  |  |  |  | **0.28** | **0.25** |  |  |  |  |  |  |  |  | **0.46** | 1.00 |  |  |  |  |  |  |
| 19.MD LC-TEC |  |  |  |  |  |  | **-0.28** | **-0.27** |  |  |  | **-0.25** |  |  |  |  |  |  | 1.00 |  |  |  |  |  |
| 20.RD LC-TEC_l |  |  |  |  |  |  |  | **-0.24** |  |  |  | **-0.25** |  |  |  |  |  |  | **0.82** | 1.00 |  |  |  |  |
| 21.RD LC-TEC_r |  |  |  |  |  |  | **-0.35** | **-0.25** | **-0.29** |  |  |  |  |  |  |  |  |  | **0.36** | **0.33** | 1.00 |  |  |  |
| 22.Neg_reappraisal |  |  |  |  |  |  |  |  |  |  |  |  |  |  |  |  |  |  |  |  |  | 1.00 |  |  |
| 23.mean LCCR |  |  |  |  |  |  |  |  |  |  |  |  |  |  |  |  |  |  |  |  |  |  | 1.00 |  |
| 24.age |  |  |  |  |  |  | **-0.44** | **-0.40** | **-0.27** |  |  | **-0.25** |  |  |  |  |  | **-0.27** | **0.46** | **0.43** | **0.42** |  |  | 1.00 |

### **Supplementary Table 7: Standardized covariance/regression estimates and fit indices for the correlational models.**

| Correlational model  (+ *covariate)* | Group | Parameters of interest | Standardized covariance/regression estimate | Model fit indices (CFI, RMSEA, SRMR) |
| --- | --- | --- | --- | --- |
| rHRV, ERn, ERp | Whole | rHRV ↔ ERn | **0.290**** | 0.980, 0.031, 0.039 |
|  |  | rHRV ↔ ERp | **-0.176**** |  |
|  |  | ERp ↔ ERn | -0.003 |  |
|  | Younger | rHRV ↔ ERn | **0.337**** | 0.965, 0.035, 0.059 |
|  |  | rHRV ↔ ERp | -0.062 |  |
|  |  | ERp ↔ ERn | -0.040 |  |
|  | Older | rHRV ↔ ERn | -0.035 |  |
|  |  | rHRV ↔ ERp | **-0.285**** |  |
|  |  | ERp ↔ ERn | 0.046 |  |
| rHRV, ERn, ERp  *age* | Whole | rHRV ↔ ERn | 0.115 | 0.987, 0.026, 0.031 |
|  |  | rHRV ↔ ERp | **-0.190**** |  |
|  |  | ERp ↔ ERn | 0.041 |  |
|  |  | Age → rHRV | **-0.255**** |  |
|  |  | Age → ERn | **-0.879**** |  |
|  |  | Age → ERp | -0.027 |  |
|  | Younger | rHRV ↔ ERn | 0.103 | 0.957, 0.039, 0.063 |
|  |  | rHRV ↔ ERp | -0.122 |  |
|  |  | ERp ↔ ERn | 0.155 |  |
|  |  | Age → rHRV | **-0.428**** |  |
|  |  | Age → ERn | **-0.698**** |  |
|  |  | Age → ERp | -0.010 |  |
|  | Older | rHRV ↔ ERn | 0.137 |  |
|  |  | rHRV ↔ ERp | **-0.282**** |  |
|  |  | ERp ↔ ERn | -0.065 |  |
|  |  | Age → rHRV | 0.097 |  |
|  |  | Age → ERn | **-0.663**** |  |
|  |  | Age → ERp | -0.117 |  |
| ΔHRV, ERn, ERp | Whole | ΔHRV ↔ ERn | -0.006 | 0.990, 0.022, 0.036 |
|  |  | ΔHRV ↔ ERp | -0.026 |  |
|  |  | ERp ↔ ERn | 0.029 |  |
| rHRV, ERposreg, ERposreac, ERbasnegaf, ERnegreac | Whole | rHRV ↔ ERposreg | **-0.212**** | 0.995, 0.023, 0.029 |
|  |  | rHRV ↔ ER_posreac | **-0.207**** |  |
|  |  | rHRV ↔ ER_basnegaf | -0.162 |  |
|  |  | rHRV ↔ ER_negreac | **-0.171**** |  |
|  | Younger | rHRV ↔ ERposreg | -0.036 | 1.000, 0.000, 0.054 |
|  |  | rHRV ↔ ER_posreac | -0.051 |  |
|  |  | rHRV ↔ ER_basnegaf | -0.054 |  |
|  |  | rHRV ↔ ER_negreac | -0.137 |  |
|  | Older | rHRV ↔ ERposreg | -0.249 |  |
|  |  | rHRV ↔ ER_posreac | -0.175 |  |
|  |  | rHRV ↔ ER_basnegaf | -0.079 |  |
|  |  | rHRV ↔ ER_negreac | -0.105 |  |
| rHRV, ERposreg, ERposreac, ERbasnegaf, ERnegreac  *age* | Whole | rHRV ↔ ERposreg | -0.079 | 0.988, 0.030, 0.031 |
|  |  | rHRV ↔ ER_posreac | -0.080 |  |
|  |  | rHRV ↔ ER_basnegaf | -0.014 |  |
|  |  | rHRV ↔ ER_negreac | -0.112 |  |
|  |  | Age → ERposreg | **0.489**** |  |
|  |  | Age → ER_posreac | **0.413**** |  |
|  |  | Age → ER_basnegaf | **0.632**** |  |
|  |  | Age → ER_negreac | **0.198**** |  |
|  | Younger | rHRV ↔ ERposreg | **0.204**** | 1.000, 0.002, 0.053 |
|  |  | rHRV ↔ ER_posreac | 0.091 |  |
|  |  | rHRV ↔ ER_basnegaf | 0.232 |  |
|  |  | rHRV ↔ ER_negreac | -0.085 |  |
|  |  | Age → ERposreg | **0.420**** |  |
|  |  | Age → ER_posreac | **0.206**** |  |
|  |  | Age → ER_basnegaf | **0.297**** |  |
|  |  | Age → ER_negreac | 0.135 |  |
|  | Older | rHRV ↔ ERposreg | **-0.2830**** |  |
|  |  | rHRV ↔ ER_posreac | -0.148 |  |
|  |  | rHRV ↔ ER_basnegaf | -0.133 |  |
|  |  | rHRV ↔ ER_negreac | -0.070 |  |
|  |  | Age → ERposreg | 0.068 |  |
|  |  | Age → ER_posreac | -0.147 |  |
|  |  | Age → ER_basnegaf | 0.175 |  |
|  |  | Age → ER_negreac | -0.146 |  |
| ΔHRV, ERposreg, ERposreac, ERbasnegaf, ERnegreac | Whole | ΔHRV ↔ ERposreg | 0.073 | 0.999 0.008 0.045 |
|  |  | ΔHRV ↔ ER_posreac | 0.059 |  |
|  |  | ΔHRV ↔ ER_basnegaf | 0.040 |  |
|  |  | ΔHRV ↔ ER_negreac | 0.106 |  |
|  | Younger | ΔHRV ↔ ERposreg | -0.054 | 1.00, 0.00, 0.05 |
|  |  | ΔHRV ↔ ER_posreac | 0.031 |  |
|  |  | ΔHRV ↔ ER_basnegaf | -0.094 |  |
|  |  | ΔHRV ↔ ER_negreac | 0.117 |  |
|  | Older | ΔHRV ↔ ERposreg | 0.188 |  |
|  |  | ΔHRV ↔ ER_posreac | 0.049 |  |
|  |  | ΔHRV ↔ ER_basnegaf | 0.125 |  |
|  |  | ΔHRV ↔ ER_negreac | 0.062 |  |

Parameters of interest were covariances (↔) between resting HRV (rHRV) or HRV reactivity (ΔHRV), ERn (‘neural’ emotion regulation), ERp (Negative Reappraisal), or the four latent emotionality factors, Positive Regulation (ERposreg), Positive Reactivity (ERposreac), Basal Negative Affect (ERbasnegaf), and Negative Reactivity (ERnegreac), +/- age regressions (→). The latent emotionality factor models were exploratory and not pre-registered. Where full metric invariance was achieved, younger and older groups were analyzed in a multigroup model where factor loadings were constrained to be equal across groups. Statistical significance of individual parameters of interest (******) were assessed by a likelihood ratio test that compared a model in which the parameter-of-interest was fixed to zero against a model in which the parameter was freely estimated (output reported in main text). Models shown included the ‘neural’ emotion regulation latent factor that comprised primary and secondary measures, as this was qualitatively similar to the model with only primary measures. The ΔHRV models did not show significant covariances, so age-adjustment was not conducted, and the first ΔHRV model did not show metric invariance, so multigroup analysis was not conducted.

### **Supplementary Figure 4: Correlational multigroup SEM model for resting HRV and four latent emotionality factors**


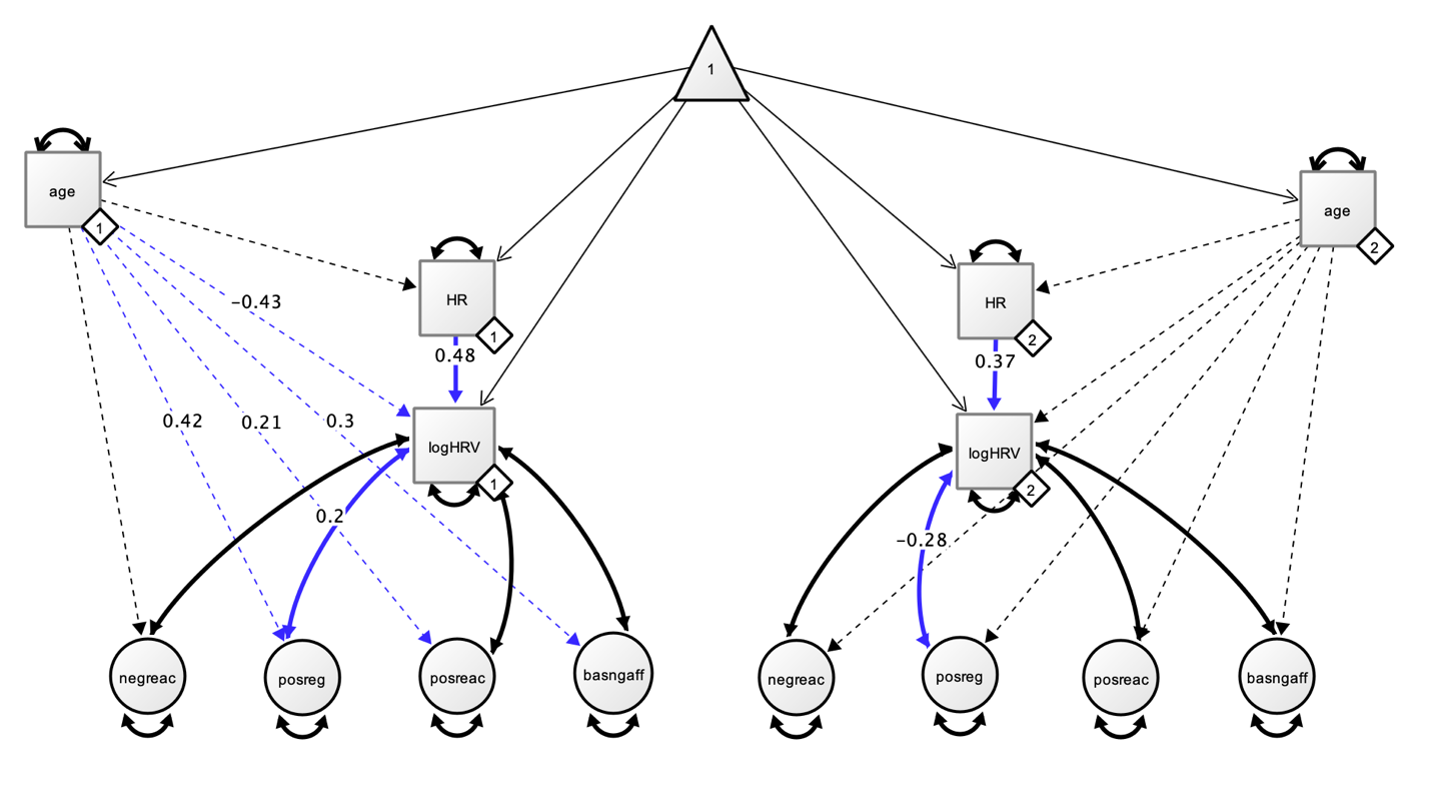


A multigroup structural equation model was used to examine covariances between resting HRV and four latent emotionality factors, which explained ERRT task scores (not shown) from the Cam-CAN dataset: Basal Negative Affect (*basngaff),* Positive Reactivity (*posreac),* Positive Regulation (*posreg),* and Negative Reactivity (*negreac*)^6^. Covariances between the latent factors are not shown to aid clarity. We formally assessed the statistical significance of individual parameters of interest using the likelihood ratio test, comparing the fit of a model with the parameter of interest freely estimated to a nested model with the same parameter fixed to zero. We also used this test to assess the statistical significance of any differences in parameters of interest between younger and older age groups by comparing a model where the parameter was constrained to be equal, to one where it was freely estimated across groups. After adjusting for age, the covariance between heart rate (HR)-adjusted resting HRV (*logHRV)* and Positive Regulation was negative in older adults (χ^2^_diff_ = 4.8845, df_diff_ = 1, p = 0.0271), and positive in younger adults (χ^2^_diff_ = 4.2962, df_diff_ = 1, p = 0.0382), and the difference in this path between groups was significant (χ^2^_diff_ = 8.536, df_diff_ = 1, p = <0.003). The constant is represented by a triangle, latent factors are represented by circles and observed variables are represented by squares/rectangles. One-headed arrows indicate regressions, double-headed arrows indicate (co)variances. Only significant standardized covariances and regressions, as formally assessed using the likelihood ratio test, are reported and shown in blue. Further information on covariance/regression estimates and model fit indices are reported in online Supplementary Table 6. Younger (left side; ♢1) and older (right side; ♢2) adult submodels are shown. Number of observations used: *N* = 364 (4 missing patterns) younger adults, *N* = 314 (4 missing patterns) older adults.

Abbreviations: Cam-CAN=Cambridge Centre for Ageing and Neuroscience; ERRT= Emotional Reactivity and Regulation Task; HR=heart rate; HRV=heart rate variability.

### **Supplementary Results 1: Analytical power for moderation analysis in cross-sectional data**

Using a web application (<https://david-baranger.shinyapps.io/InteractionPoweR_analytic/>)^7^, we conducted post-hoc analytic power estimations for a cross-sectional interaction (moderation) analysis using effects observed in the dataset of interest. We calculated that the older adult sample (n=314) would provide only 44% power to detect a significant (i.e., alpha=0.05) interaction effect. As our hypothesis was specific to the older adult (versus younger adult) subgroup and these post-hoc power calculations were also likely to be overestimates, we did not conduct a moderation analysis.

**REFERENCES**

1. Taylor JR, Williams N, Cusack R, et al. The Cambridge Centre for Ageing and Neuroscience (Cam-CAN) data repository: Structural and functional MRI, MEG, and cognitive data from a cross-sectional adult lifespan sample. *Neuroimage*. 2017;144(Pt B):262-269.

2. Allen J, Murray A. Age-related changes in peripheral pulse timing characteristics at the ears, fingers and toes. *J Hum Hypertens*. 2002;16(10):711-717.

3. Trivedi NS, Ghouri AF, Shah NK, Lai E, Barker SJ. Effects of motion, ambient light, and hypoperfusion on pulse oximeter function. *J Clin Anesth*. 1997;9(3):179-183.

4. Kievit RA, Brandmaier AM, Ziegler G, et al. Developmental cognitive neuroscience using latent change score models: A tutorial and applications. *Dev Cogn Neurosci*. Published online November 22, 2017. doi:10.1016/j.dcn.2017.11.007

5. Mather M, Carstensen LL. Aging and motivated cognition: the positivity effect in attention and memory. *Trends Cogn Sci*. 2005;9(10):496-502.

6. Stretton J, Schweizer S, Dalgleish T. Age-Related Enhancements in Positive Emotionality across The Life Span: Structural Equation Modeling of Brain and Behavior. *J Neurosci*. 2022;42(16):3461-3472.

7. Baranger DAA, Finsaas MC, Goldstein BL, Vize CE, Lynam DR, Olino TM. Tutorial: Power Analyses for Interaction Effects in Cross-Sectional Regressions. *Advances in Methods and Practices in Psychological Science*. 2023;6(3):25152459231187532.
